# Impairing the production of ribosomal RNA activates mammalian target of rapamycin complex 1 signalling and downstream translation factors

**DOI:** 10.1093/nar/gku130

**Published:** 2014-02-13

**Authors:** Rui Liu, Valentina Iadevaia, Julien Averous, Peter M. Taylor, Ze Zhang, Christopher G. Proud

**Affiliations:** ^1^Centre for Biological Sciences, University of Southampton, Highfield Campus, Southampton SO17 1BJ, UK, ^2^Centre de Recherche en Nutrition Humaine, INRA Clermont-Theix, Unité de Nutrition Humaine, 63122 Ceyrat, France and ^3^Division of Molecular Physiology, James Black Centre, College of Life Sciences, University of Dundee, Dundee, DD1 5EH, UK

## Abstract

Ribosome biogenesis is a key process for maintaining protein synthetic capacity in dividing or growing cells, and requires coordinated production of ribosomal proteins and ribosomal RNA (rRNA), including the processing of the latter. Signalling through mammalian target of rapamycin complex 1 (mTORC1) activates all these processes. Here, we show that, in human cells, impaired rRNA processing, caused by expressing an interfering mutant of BOP1 or by knocking down components of the PeBoW complex elicits activation of mTORC1 signalling. This leads to enhanced phosphorylation of its substrates S6K1 and 4E-BP1, and stimulation of proteins involved in translation initiation and elongation. In particular, we observe both inactivation and downregulation of the eukaryotic elongation factor 2 kinase, which normally inhibits translation elongation. The latter effect involves decreased expression of the *eEF2K* mRNA. The mRNAs for ribosomal proteins, whose translation is positively regulated by mTORC1 signalling, also remain associated with ribosomes. Therefore, our data demonstrate that disrupting rRNA production activates mTORC1 signalling to enhance the efficiency of the translational machinery, likely to help compensate for impaired ribosome production.

## INTRODUCTION

Ribosome biogenesis, the production of new ribosomes, plays key roles in cell growth and division by providing increased capacity for protein synthesis (1,2). The transcription of the major ribosomal RNAs (rRNAs) and their processing, along with the assembly of new ribosomal subunits, occur within nuclear regions termed nucleoli (3).

The importance of enhanced ribosome biogenesis for cell proliferation is exemplified by the early observation that nucleolar morphology is markedly altered in cancer cells (4), reflecting their faster rates of ribosome production. It is now widely recognized that increased ribosome biogenesis is of key importance in cancer (4) and other disorders of cell proliferation or growth [such as cardiac hypertrophy (5,6)]. Ribosome biogenesis requires the coordinated synthesis of four rRNAs and >80 ribosomal proteins (RPs). It consumes large amounts of metabolic energy, amino acids and ribonucleotides. The mammalian target of rapamycin complex 1 (mTORC1, a protein kinase) positively regulates the transcription and processing of rDNA and the translation of the mRNAs for RPs (2,7).

mTORC1 contains mTOR (the catalytic subunit) and raptor, a scaffold protein that recruits substrates for phosphorylation by mTOR. The best-known mTORC1 substrates are the RPs S6 kinases (S6K1/2) and the eukaryotic initiation factor 4E (eIF4E)-binding proteins (4E-BPs) (8).

A number of studies have revealed roles for mTOR signalling in rRNA synthesis in yeast and in mammalian cells [reviewed in (2,9,10)]. The 5.8S, 18S and 28S rRNAs are made by RNA polymerase I (Pol I), while the 5S rRNA is made by Pol III. mTORC1 promotes the activities of Pol I and Pol III, as revealed by the inhibitory effects of rapamycin, a specific inhibitor of mTORC1 (2). However, rapamycin does not inhibit all the functions of mTORC1, as exemplified by the effects of compounds such as PP242, which directly inhibit mTOR’s kinase activity (11–13). A second, distinct mTOR complex, mTORC2, phosphorylates several protein kinases, including protein kinase B [PKB, also termed Akt (14)]. In turn, PKB, whose activity is stimulated by insulin, contributes to the activation of mTORC1 by phosphorylation and inactivation of tuberous sclerosis 2 (TSC2), a negative regulator of mTORC1 [reviewed (15)].

One mechanism by which mTORC1 may promote rRNA transcription through S6K1-dependent control of Pol I, which positively regulates rRNA synthesis as shown by the finding that it can rescue Pol I-mediated transcription from inhibition by rapamycin (16), perhaps via the regulation of the Pol I transcription regulatory component UBF [upstream binding factor; (17)] by S6K1. However, the exact mechanisms by which mTORC1 or S6K1 control Pol I remain to be clarified.

Pol I generates a single rRNA precursor, the 47S pre-rRNA, which is processed to yield the mature 5.8S, 18S and 28S rRNAs. Pre-rRNA maturation involves a large number of components including the PeBoW complex, comprising the proteins PES1, BOP1 and WDR12 (18). The importance of this complex for rRNA processing is illustrated, for example, by the fact that a truncated version of BOP1 (BOP1Δ) interferes with processing of the 32S pre-rRNA intermediate into 28S rRNA (19). We previously showed that rapamycin or the mTOR kinase inhibitor AZD8055 (20) interferes with pre-RNA processing (7), although the mechanism(s) that link mTORC1 to this process remain unclear.

mTORC1 signalling also contributes to the activation of ribosome biogenesis by promoting the translation of the mRNAs encoding RPs. They contain a 5′-terminal tract of pyrimidines (5′-TOP) that confers control by mTORC1 of the recruitment of ribosomes on to these messages, which also include mRNAs encoding all translation, elongation and several initiation factors (21).

In addition to enhancing the protein synthetic capacity of cells by promoting ribosome production, mTORC1 also promotes the translational efficiency of cells, by activating translation initiation by alleviating the inhibition of eukaryotic initiation factor eIF4E by 4E-BPs and translation elongation via the inactivation of eukaryotic elongation factor 2 kinase [eEF2K; a substrate for S6K1 (22)] and the resulting dephosphorylation and activation of eEF2 (23). eEF2K can also be controlled through additional mechanisms, some of which are mediated by mTORC1 (23,24).

Given the importance of ribosome biogenesis, both in normal cell physiology and in disease states, it is important to gain a much better understanding of its control, e.g. by mTORC1. Furthermore, it is not clear whether and how ribosome biogenesis is coupled to the rate of protein synthesis or vice versa.

Here, we have asked how mammalian cells respond to impaired production of rRNA. We show that interference with rRNA processing, and therefore with the production of mature rRNAs for ribosome biogenesis, leads to activation of S6K1 and the disinhibition of the initiation and elongation stages of mRNA translation. Thus, these data reveal a feedback mechanism in mammalian cells, whereby defects in rRNA biosynthesis cause the activation of mTORC1 signalling and of components involved in rRNA transcription and mRNA translation. In addition to helping promote ribosome biogenesis in response to defective rRNA maturation, this mechanism likely acts to allow cells to maximize the efficiency of the existing translational machinery under conditions where rRNA synthesis is impaired.

## MATERIALS AND METHODS

### Chemicals and antibodies

Doxycycline was purchased from Fisher, MG132 from Calbiochem, rapamycin from Merck, PP242 from Sigma-Aldrich, AZD6244 from Selleck and PF4708671 from Tocris. The anti-myc and anti-B23 antibodies were purchased from Sigma-Aldrich; anti-HA was from Roche Applied Sciences; anti-raptor, rictor, 4E-BP1, PKB and their phosphorylation site-specific antibodies were from Cell Signalling Technology; anti-tubulin, anti-RPL11, anti-RPL28 and anti-RPS6 were from Santa Cruz Biotechnology. Secondary antibodies were from Li-Cor Biosciences. Anti-LAMP2 was purchase from Abcam.

### Vectors and oligonucleotides

The pEBG-6P empty vector was derived from pEBG2T as described in (25). pGEX-6P-1 was purchased from Amersham, pCDNA5FRT and pOG44 were from Invitrogen. pGEX-6P-4E-BP1 and its mutation were described previously (26). The cDNA clone for human BOP1 was purchased from Origene and subcloned into appropriate vectors for expression as a glutathione S-transferase (GST) fusion protein in bacteria (pGEX6p-1, between the EcoR1 and Not1 sites) or mammalian cells by transient transfection (pEBG6P-1, between the Spe1 and Cla1 sites) or in a doxycycline-inducible manner (pCNDA5FRT, HindIII + XhoI). The truncation mutant of BOP1 was created by polymerase chain reaction (PCR) and cloned similarly. All vectors were fully sequenced after construction or mutagenesis.

siRNA oligonucleotides for BOP1, PES1, WDR12 and scrambled siRNA were from (27). The siRNAs used were as follows: BOP1-1, UCGUGCUGAAGUCAACAGAdTdT; BOP1-2, dCCAAGAAGCUGAUGCCCAAdTdT; WDR12-2, CGUACGUUUCCGUGGGCAAdTdT; PES1, CCAGAGGACCUAAGUGUGAdTdT; scrambled, UUCUCCGAACGUGUCACGUdTdT. The expression vectors for V5-tagged Rag mutants were described in (28).

### Immunoprecipitation and S6K1 kinase assay

Ten microlitres of protein G-Sepharose beads and 0.5 µg of anti-HA high-affinity antibody were incubated together at 4°C for 2 h, then unbound antibody was washed away by lysis buffer. One milligram of crude lysate from transfected cells was added into protein G-Sepharose slurry. After 1 h of incubation at 4°C, the protein G-Sepharose beads were washed twice with lysis buffer.

Purified HA-S6K1 was immunoprecipitated from different groups of cells. Kinase reactions were performed by incubating HA-S6K1 with 5 nmol ‘Crosstide’ substrate, 1.25 nmol cold ATP, 0.3 μCi of [γ-^32^P]ATP and kinase buffer in a final volume of 20 µl. Kinase reactions were performed at 30°C for 15 min, transferred samples onto filters and then placed into 150 mM phosphoric acid. The filters were washed three times with phosphoric acid. Radioactivity associated with the filters was determined using a Čerenkov programme.

### Cell lysis and western blotting

Cells were lysed directly on the plate by the addition of cold lysis buffer [50 mM Tris–HCl, 50 mM β-glycerophosphate, 1 mM ethylene glycol tetraacetic acid (EGTA), 1 mM ethylenediaminetetraacetic acid (EDTA) and 1% (v/v) Triton X-100]. β-Mercaptoethanol, Na_3_VO_4_ and protease inhibitor cocktail were added to the lysis buffer just before use. Lysates were centrifuged at 4°C at maximum speed for 10 min and then the supernatant was collected. Protein concentrations were determined by Bradford reagent (Bio-Rad). Cell lysates or immunoprecipitated samples were heated at 95°C for 5 min in sample buffer [62.5 mM Tris–HCl, 7% (w/v) sodium dodecyl sulfate (SDS), 20% (w/v) sucrose and 0.01% (w/v) bromophenol blue] and subjected to polyacrylamide gel electrophoresis (PAGE) and electrophoretic transfer to nitrocellulose/polyvinylidene difluoride membranes. Membranes were then blocked in phosphate-buffered saline (PBS)-Tween 20 containing 5% (w/v) skimmed milk powder for 30 min at room temperature. Membranes were probed with the indicated primary antibody overnight at 4°C. After incubation with fluorescently tagged secondary antibody, signals were scanned using a Li-Cor Odyssey imaging system.

### Measurement of protein synthesis rates

T-REx cells were starved in methionine-free medium (Biosera DMEM SM-D3666) for 1 h and incubated with [^35^S]methionine (Perkin Elmer; 10 μCi/ml) for 1 h at 37°C. After this incubation, the medium was removed completely and the cells were washed in ice-cold PBS and lysed in 50 mM Tris–HCl (pH 7.5), 50 mM β-glycerophosphate, 100 mM NaCl, 0.5 mM EDTA and 1% Triton X-100. The protein concentrations were then quantified using the Bradford. Lysate (5 µl) was applied to 3MM filter papers (Whatman), which were washed twice with 5% (w/v) trichloracetic acid and once in acetone (100%). Incorporated radioactivity was measured by scintillation counting.

### eEF2 kinase assay

Recombinant eEF2 (1 μg) was incubated with Ca^2+^/CaM (0.2 mM/10 μg/ml) and [γ-^32^P]ATP (0.1 mM, 1 μCi per reaction) in 30 μl of reaction buffer containing [γ-^32^P]ATP [final concentration 0.1 mM, 1 μCi per reaction; 50 mM 3-(N-morpholino)propanesulfonic acid, pH 7.0 (unless pH stated otherwise), 20 μg/ml CaM (where present), 5 mM MgCl_2_, 14 mM β-mercaptoethanol, 0.67 mM CaCl_2_, 2 mM EDTA, 0.4 mM EGTA, 1 mM benzamidine-HCl and 1 mM each of leupeptin, pepstatin and antipain] at 30°C for 15 min. SDS-PAGE was performed followed by staining with coomassie brilliant blue, the gels were placed into destain/fixing solution [50% (v/v) methanol, 10% (v/v) acetic acid]. Radioactivity was detected using phosphor imager screen (Typhoon, GE Healthcare).

### Polysome analysis and northern blot analyses

T-REx cells had been washed twice with ice-cold PBS and lysed directly on the plate with 300 µl of lysis buffer (10 mM NaCl, 10 mM MgCl_2_, 10 mM Tris–HCl, pH 7.5, 1% Triton-X100, 1% sodium deoxycholate, 36 U/ml RNase inhibitor (Promega) and 1 mM dithiothreitol). Sucrose gradient density centrifugation was performed as recently described (7). RNA was isolated by proteinase K method (7) or proteins were precipitated from each fraction using trichloroacetic acid (TCA) at a final concentration of 10% (w/v). Radiolabelled probes were prepared by random priming as described earlier (7).

### Ribosome fractionation

The cells lysed in tetranitromethane buffer were centrifuged in a Beckman T70.1 rotor for 2 h at 100 000×*g* on a 25% (v/v) sucrose cushion. After centrifugation, two fractions were obtained: fraction P (pellet), which includes polysomes and ribosomal subunits, and fraction S (supernatant), which includes cytoplasmic proteins. The proteins from fraction S were precipitated with 10% TCA and the proteins in fraction P were directly resuspended in SDS-PAGE loading buffer.

### Proliferation assay

T-REx cells (non-induced or induced with 1 μg/ml doxycycline) were split in triplicate in six-well plates in DMEMmedium containing 10% foetal bovine serum. Every 48 h, cells were counted with a Neubauer counting chamber. 3-(4,5-Dimethylthiazol-2-yl)-2,5-diphenyltetrazolium bromide(MTT) assay (M2003, Sigma-Aldrich) was performed as recommended by the manufacturer.

### RNA preparation, northern blot and pre-rRNA species

Total RNA was extracted by the proteinase K method (7). For northern blot analysis, RNA (2 µg) was run on 1.5% agarose-formaldehyde gels and transferred to GeneScreen Plus membrane (PerkinElmer Life Sciences) for 15 h at 4°C. Northern blotting was performed as recommended by the manufacturer. Oligonucleotides were end-labelled with [α-^32^P]ATP and T4 polynucleotide kinase (BioLabs M0201S) for 30 min at 37°C. Membranes were hybridized overnight in hybridization buffer (6× saline–sodium phosphate–EDTA (SSPE), 5× Denhardt`s solution, 0.1 mg/ml fish sperm DNA and 1% SDS). Washing was performed in 2× SSPE, 1% SDS at 65°C for 30 min.

The pre-rRNA species were detected using oligodeoxynucleotide complementary to the human rDNA: 5′ITS2 (5′-CGCACCCCGAGGAGCCCGGAGGCACCCCCGG-3′). Membranes were exposed to storage phosphor screen (GE-Healthcare) and analysed using a phosphorimager (STORM, GE Healthcare). Quantitation of northern blots was performed using the Image-Quant software (Amersham Biosciences).

### RT-Real time PCR amplification analysis

Total RNA (2 µg) was extracted by the proteinase K method (7) and then subjected to ImProm-IItm Reverse Transcription System (A3800 Promega) with oligo(dT)_15_ and random primers following the manufacturer’s protocol. Subsequently, the real-time PCR was performed using specific primers (PrimerDesign) for eukaryotic elongation factor-2 kinase (eEF2K) (5′-CCAGCCAAGACTTCAGTGTT-3′; 5′-ATTTTACCTGCTTCATTGTTCATTTAA-3′), β-actin (5′-CATTGGCAATGAGCGGTTC-3′; 5′-CCACGTCACACTTCATGATGG-3′) and p21 (5′-GCAGACCAGCATGACAGATTT-3′; 5′-AAGATGTAGAGCGGGCCTTT-3′). The samples were analysed in triplicate with SYBR Green dye (Primer Design mix) on an ABI StepOnePlus quantitative PCR instrument (Applied Biosystems). The comparative Ct method was used to measure amplification of eEF2k mRNAs versus β-actin.

### Amino acid levels

Levels of amino acids in cell lysates were determined as described (29).

### Chromatin cross-linking and immunoprecipitation—ChIP

T-REx cells were induced for 15 h with 1 µg/ml doxycycline. Cross-linking was performed by adding 1% formaldehyde for 10 min at room temperature. The cells were lysed in 350 µl of lysis buffer (1% SDS, 10 mM EDTA, 50 mM Tris–HCl, pH 8.1) and sonicated seven times for 20 s. The ChIP antibody used was specific for UBF (Santa Cruz, #9131). For normalization, an input of DNA was prepared and subject to qPCR analysis using primers specific for rDNA region (5′-CGACGACCCATTCGAACGTCT-3′; 5′-CTCTCCGGAATCGAACCCTGA-3′). The samples were analysed with SYBR Green dye (Primer Design mix) on an ABI StepOnePlus quantitative PCR instrument (Applied Biosystems).

### Immunostaining

Cell imaging was performed with a confocal laser-scanning microscope (Leica SP5). Chambered coverslips were used for immunostaining. Cells were fixed with 4% (v/v) para-formaldehyde for 5 min and permeabilized with PBS 0.1% Triton X100 for 2 min. Cells were rinsed three times with PBS and then incubated with PBS containing 0.5% (w/v) bovine serum albumin (BSA) for 1 h at room temperature. Primary antibodies, diluted at 1/200 in PBS containing 0.5% BSA, were incubated at 4°C for 16 h. Secondary antibodies, diluted at 1/500 in PBS containing 0.5% BSA, were incubated at room temperature for 1 h in the dark.

The Costes' approach was used to quantify the protein–protein co-localization in cells using MBF ImageJ. This allows for the calculation of Pearson's correlation coefficient R(obs) between n images. In theory, an R(obs) of +1 would represent a perfect pixel correlation between twochannels in an image, whereas an R(obs) of 0 represent a random correlation and therefore no co-localization.

### Cells inducibly expressing BOP1Δ

Cell lines inducibly expressing BOP1Δ mutant was generated by using Invitrogen’s FLP/FRT system according to the manufacturer’s instructions.

### RNA preparation and biotinylation of 4-TU-labelled RNA

The procedure for labelling newly synthesized RNA was described in (7). The 28S, 18S and p21 primers were purchased from PrimerDesign.

### Subcellular fractionation

T-REx cells were washed twice with ice-cold PBS and resuspended in cold buffer A (10 mM Tris–HCl, pH 7.5, 10 mM NaCl, 3 mM MgCl_2_, 0.05% of Nonidet P-40 and protease inhibitor). The cell lysates were centrifuged at 100 rcf for 5 min at 4°C. After removal of the supernatant (the cytoplasmic fraction), the resulting pellet (the nuclear fraction) was washed five to six times with buffer A and resuspended in cold buffer B [20 mM Tris–HCl, pH 7.5, 420 mM NaCl, 1.5 mM MgCl_2_, 1 mM EDTA, 1 mM EGTA, 20% (v/v) glycerol, 1% (v/v) Triton and protease inhibitors]. The nuclear extract was incubated for 1 h at 4°C on a rotating tube mixer. It was then centrifuged for 10 min at 15 000 rcf at 4°C, after which the supernatant was carefully collected.

## RESULTS

### Expression of BOP1Δ promotes activation of S6K1

**Figure 1.**
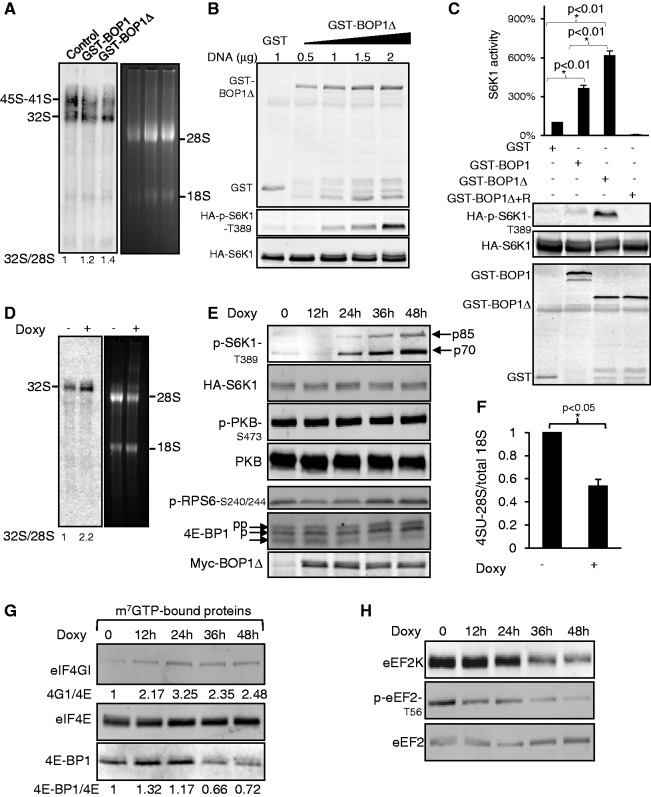
Expressing BOP1Δ elicits activation of S6K1. (**A**) Northern blot hybridization of total RNA. HEK293 cells were transiently transfected with 1 µg of GST-BOP1, GST-BOP1Δ or GST vectors. After 48 h, total RNA was extracted and subjected to northern blot analysis using a probe specific for the precursors corresponding to the 5′-end of ITS2. The numbers below the lanes refer to the quantification for the 32S normalized to the signal for 28S (control value set at 1). (**B**) HEK293 cells were transfected with 0.5 µg of HA-S6K1 DNA and series of concentrations of GST-BOP1Δ DNA. After 48 h, cells were harvested, and 20 µg of lysate was analysed by western blots using the indicated antisera. (**C**) HEK293 cells were transfected with 0.5 µg of HA-S6K1 DNA and 1 µg of GST-BOP1, GST-BOP1Δ or GST DNA as indicated; 35 h later, where indicated, cells were treated with rapamycin (100 nM) for 45 min. HA-S6K1 was immunoprecipitated from 1 mg of total lysate. Immunoprecipitates were aliquoted into three equal portions: two were analysed by western blot to verify the IP efficiency, while the third was used for S6K1 assay. Total lysates were analysed by western blot to confirm expression of GST-tagged proteins. (**D**) Expression of BOP1Δ was induced by treating T-REx cells for 15 h with 1 µg/ml doxycycline. Total RNA was extracted and subjected to northern blot to detect the pre-rRNA (using the ITS2 probes).The numbers below the lanes refer to the quantification for 32S: 28S as in panel (A). (**E**) T-REx cells were treated with doxycycline for the indicated times, harvested and 20 µg of lysate was analysed by western blots; p85 and p70 denote the different isoforms of S6K1, which are observed here. (**F**) T-REx cells were treated with 1 µg/ml doxycycline for 15 h before addition of 4-SU. Total RNA was extracted after 4 h and processed to measure levels of labelled 28S rRNA, relative to total 18S rRNA, as described in the ‘Materials and Methods’ section. (**G**) Cell lysates were subjected to affinity chromatography on m^7^GTP–Sepharose and the bound material was analysed by western blot. Numbers show the quantification for eIF4G:eIF4E and 4E-BP1:eIF4E binding from a typical experiment (untreated cells value set at 1). (**H**) Cells were treated with doxycycline as in panels E and G. Twenty micrograms of lysate was analysed by western blot. Significance was determined by *t*-test.

The truncation mutant of mouse BOP1 lacking residues 1–250 has been shown to interfere with processing of the 47S rRNA precursor (30). To explore the effects of interfering with ribosome biogenesis on the signalling pathways involved in this process and the control of mRNA translation, we tested the effect of expressing the equivalent truncation of human BOP1 (lacking residues 1–264) in HEK293 cells. We initially used GST-tagged BOP1 or BOP1Δ because this tag allows us to perform diverse types of experiments. As reported for mouse cells (30), expressing BOP1Δ interferes with processing of pre-rRNA in human cells, as shown by accumulation of 32S pre-rRNA relative to the 28S (Figure 1A).

To examine the cellular response to interference with rRNA processing, we monitored the activation state of the mTORC1 pathway, which promotes both ribosome biogenesis and mRNA translation (2,23), by studying the phosphorylation of S6 kinase 1 (S6K1). Expressing GST-BOP1Δ caused a dose-dependent increase in phosphorylation of S6K1 at Thr389, a key residue for its activation, which is phosphorylated by mTORC1 (31) (Figure 1B). At equivalent levels of expression, BOP1Δ stimulated S6K1 phosphorylation more strongly than full-length BOP1 (Figure 1C and Supplementary Figure S1A). Immunoprecipitation of the co-expressed HA-S6K1, followed by a kinase assay, confirmed that expression of BOP1Δ, and to a lesser extent, BOP1, did activate S6K1 (Figure 1C). The activation of S6K1 caused by expressing by BOP1Δ was completely blocked by rapamycin, indicating that, as expected, it is mediated via mTORC1 in this setting (Figure 1C).

It was possible that BOP1Δ activated mTORC1 independently of the mechanisms that normally control its activity: mTORC1 signalling normally requires amino acids and is further activated by, for example, insulin [see, e.g. (32)]. BOP1Δ markedly potentiated the phosphorylation of S6K1 observed in the presence of amino acids/insulin (Supplementary Figure S1B). However, S6K1 phosphorylation was still stimulated by amino acids, and was further enhanced by insulin, indicating that BOP1Δ enhances S6K1 activation without bypassing the normal control of mTORC1 signalling.

### Effects of inducible expression of BOP1Δ

To obviate the limitations imposed by transient transfection, we created a stable cell line in which BOP1Δ can be expressed in an inducible manner, and replaced the GST tag with the smaller myc tag. Like endogenous BOP1 (7), myc-BOP1Δ was primarily nuclear (Supplementary Figure S2A). Induction of myc-BOP1Δ interfered with rRNA processing, increased the levels of the 32S rRNA precursor (Figure 1D) and elicited phosphorylation of S6K1 (Supplementary Figure S2B) in a time-dependent manner (Figure 1E). Phosphorylation of PKB at Ser473 was unaffected (Figure 1E), indicating that BOP1Δ does not alter mTORC2 function. Induction of myc-BOP1Δ also enhanced the transcription of p21 (Supplementary Figure S2C), a gene that responds to p53, which is stabilized in response to ribosomal stress (33). Induction of myc-BOP1Δ also impaired cell proliferation (cell number; Supplementary Figure S2D), accumulation of cell mass (MTT assay, Supplementary Figure S2E), impairment of UBF binding to the Pol I promoter (Supplementary Figure S2F). Therefore, the inducible expression of myc-BOP1Δ exerts similar effects to those reported for transient expression of BOP1Δ (19,30). In addition, we studied the distribution of the nucleolar protein B23 (Supplementary Figure S2G) following knock-down of BOP1. RNA-interference-mediated knock-down of BOP1 caused an increase of localization in the nucleoplasm of B23 similar to the actinomycin D treatment shown previously (7). These data support the conclusion that defects in BOP1 cause nucleolar stress.

As S6K1 has been shown to positively regulate rRNA transcription [reviewed in (9)], this could constitute a ‘rescue’ mechanism to promote rRNA synthesis in response to inadequate ribosome production. To study rRNA synthesis, we used a non-radioactive labelling method [whereby new rRNA is tagged with 4-thiouracil, 4SU (7)]. The data (Figure 1F) reveal a decrease in the accumulation of new labelled rRNA in cells expressing BOP1Δ perhaps because impairing rRNA processing destabilizes new rRNA, in an analogous way to the effect of rapamycin, which also interferes with processing (7). This is consistent with the observation that expressing BOP1Δ markedly impaired the association of UBF with rDNA (Supplementary Figure S2F). Thus, although mTORC1 signalling and the S6Ks are implicated in promoting Pol I transcription (9,17), the activation of mTORC1 signalling induced by BOP1Δ is insufficient to promote accumulation of new rRNA in the face of impaired pre-rRNA processing. It is not clear whether the impaired accumulation of new rRNA is only due to inhibition of transcription, or whether increased decay of new pre-rRNA may also contribute to this.

### Inducible expression of BOP1Δ causes activation of translation initiation and elongation factors

The translational repressor protein, 4E-BP1, undergoes phosphorylation by mTORC1 at multiple sites, retarding its mobility on SDS-PAGE. In cells expressing BOP1Δ, 4E-BP1 underwent an upward mobility shift (Figure 1E), indicating its increased phosphorylation (N.B. all three species of 4E-BP1 observed in this figure are phosphorylated on T37/46; they differ in their phosphorylation at other sites). This further supports the conclusion that expressing BOP1Δ enhances mTORC1 signalling. When eIF4E was isolated from cell lysates (by affinity chromatography on m^7^GTP-Sepharose beads), we observed partial loss of 4E-BP1 associated with eIF4E concomitant with its increased phosphorylation (Figure 1G). Conversely, as expected, the association of eIF4E with eIF4G increased, indicating enhanced formation of cap-binding initiation complexes.

S6K1 promotes translation elongation by phosphorylating and inactivating eEF2 kinase (eEF2K), which phosphorylates and inhibits eEF2 (22). Consistent with this, expression of BOP1Δ led to dephosphorylation of eEF2 (Figure 1H). Expressing BOP1Δ also led to a decrease in the expression of eEF2K (Figure 1H). However, the dephosphorylation of eEF2 preceded the fall in eEF2K levels, suggesting that the early stages of this response may be due to inactivation of eEF2K, a point which is explored in detail below. The dephosphorylation of eEF2 and the disinhibition of eIF4E seen in Figure 1G show that BOP1Δ expression promotes both the initiation and elongation stages of mRNA translation. This amounts to an increase in the efficiency of the translational machinery in the face of insufficient ribosome production.

### Other ways of interfering with rRNA production also lead to phosphorylation of S6K1 and the dephosphorylation of eEF2

**Figure 2.**
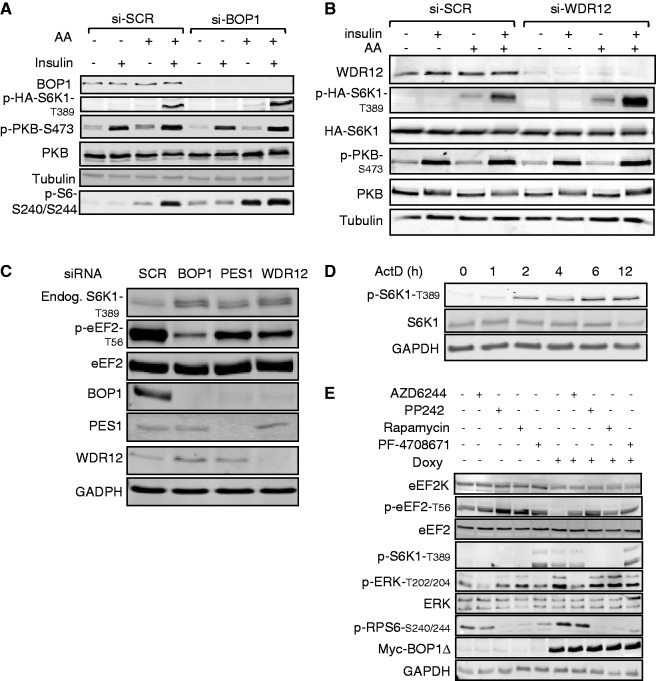
Interference with the PeBoW complex regulates effectors of mTORC1. (**A** and **B**) HEK293 cells were transfected with 0.5 µg of HA-S6K1 DNA; 24 h later, cells were transfected with scrambled siRNA or siRNA against BOP1 (20 nM) as indicated. After 48 h, cells were starved overnight of serum and in some cases, subsequently starved of amino acids (1 h). Some cells were then stimulated with insulin (100 nM; 30 min). Cell lysates were subjected to western blot. (**C**) Cells were cultured in complete medium and transfected with scrambled siRNA or siRNA against BOP1, WDR12 or PES1 (20 nM). After 72 h, cells were lysed and samples subjected to western blot. (**D**) HEK293 cells were treated with actinomycin D (20 ng/ml) for different times. Cells were then lysed and samples subjected to western analysis. (**E**) T-REx cells expressing BOP1Δ were cultured in complete medium with/without doxycycline for 48 h. The cells were treated with 1 µM PP242, 100 nM rapamycin, 1 µM AZD6244 or 10 µM PF-4708671 for 1 h. Cells were lysed and 20 µg of lysate proteins were used for western blot analysis.

To assess whether the effect of BOP1Δ was due specifically to this truncated protein or interference with rRNA production, we examined the effects of other treatments designed to interfere with this complex. Knock-down of WDR12 or Pes1 interferes with rRNA processing (27,34). Knocking down BOP1, WDR12 or, to a lesser extent, PES1 also increased the phosphorylation of HA-S6K1 (Figure 2A and B) or endogenous S6K1 (Figure 2C and Supplementary Figure S3A–C) at T389. In contrast, knocking down WDR12 or BOP1 did not affect the phosphorylation of PKB at S473 (Figure 2A and B and Supplementary Figure S3A and B), indicating it does not influence mTORC2 function. The phosphorylation of HA-S6K1 induced by knocking down BOP1 or WDR12 required the presence of both amino acids and insulin (Figure 2A and B). Two different siRNAs directed at BOP1 had similar effects (Supplementary Figure S3C). Thus, any of these ways of impairing PeBoW function leads to enhanced phosphorylation of S6K1 and dephosphorylation of eEF2, consistent with enhanced mTORC1 signalling.

It was important to examine whether other ways of disrupting ribosome biogenesis induce a similar effect; we therefore tested the effect of actinomycin D at low concentrations, which selectively inhibit Pol I [rather than the higher concentrations used earlier (35), which inhibit other RNA polymerases]. This also elicited the gradual phosphorylation of S6K1 (Figure 2D). Thus, distinct ways of interfering with rRNA production have a common outcome, i.e. activation of mTORC1 signalling and increased S6K1 phosphorylation.

### Induction of BOP1Δ interferes with polyribosome formation and protein synthesis

**Figure 3.**
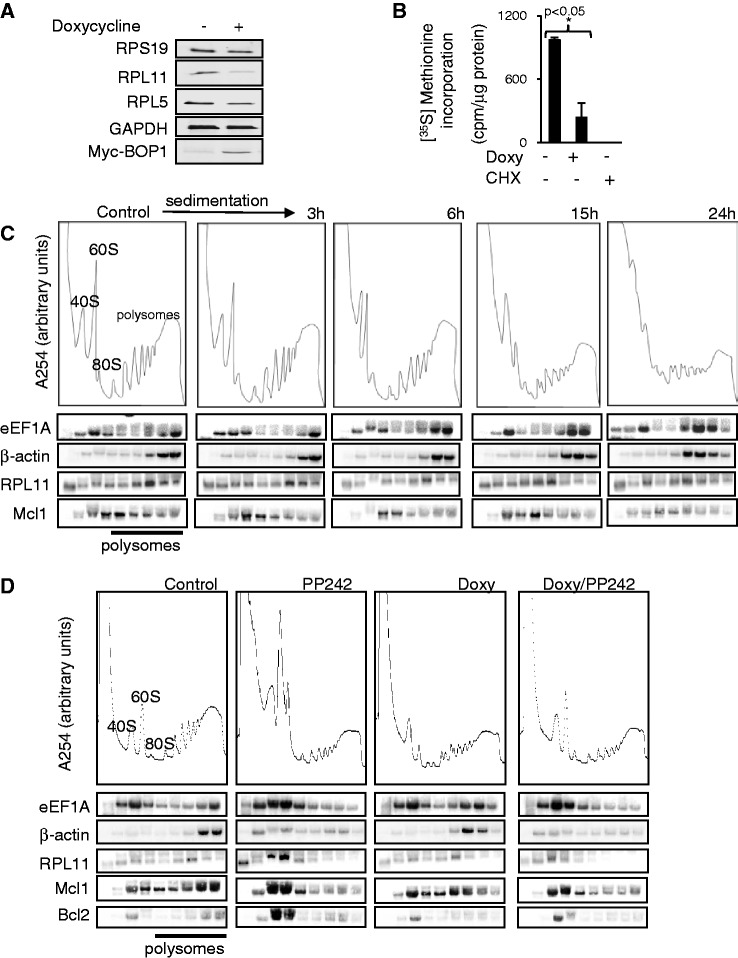
5′-TOP mRNAs are translationally active in cells expressing BOP1Δ. (**A**) T-REx cells were treated for 15 h with 1 µg/ml doxycycline and samples subjected to western blot. (**B**) The rate of protein synthesis was then analysed by incorporation of [^35^S]methionine. Data are derived from three independent experiments. Significance was determined by Student’s *t*-test. (**C**) The T-REx cells were induced for the indicated times with 1 µg/ml doxycycline and lysates were fractionated on sucrose-density gradients. Positions of the 40S, 60S and 80S ribosomal particles and polysomes are shown; absorbance values at 254 nm are in arbitrary units and are on the same scale for each panel. RNA was extracted from each fraction and subjected to northern blot using probes specific for eEF1A, β-actin, RPL11 and Mcl1 mRNAs. (**D**) The T-REx cells were treated with 1 µg/ml doxycycline for 48 h and with 1 µM PP242 for 15 h. The lysate was fractionated on sucrose-density gradients. RNA was extracted from each fraction and subjected to northern blot using probes specific for eEF1A, β-actin, RPL11, Bcl2 and Mcl1 mRNAs.

Although expression of BOP1Δ enhances mTORC1 signalling, it also interferes with ribosome biogenesis. In cells overexpressing BOP1Δ, levels of the 60S proteins RPL5, RPL11 and RPL28 declined (Figure 3A and Supplementary Figure S3D), perhaps owing to their rapid degradation (36) because they cannot be incorporated into ribosomal particles owing to insufficient mature 28S rRNA, although expressing BOP1Δ only caused a slight decrease in the 40S component RPS19. Levels of 40S subunit proteins also declined, but to a lesser extent (Supplementary Figure S3D). Given the decrease in the levels of components of the 60S subunit, we anticipated that overall protein synthesis might decrease. Consistent with this idea, induction of BOP1Δ for 15 h decreased the rate of protein synthesis by ∼75% (Figure 3B).

To further assess the impact of interfering with the PeBoW complex on the translational machinery, we induced BOP1Δ for various times, and then fractionated the resulting cell lysates on sucrose density gradients. A marked decrease in 60S subunits (Figure 3C) was already evident by 3 h, reflecting their impaired production. There was little effect on the profile of polysomal particles at 3 or 6 h, apart from the appearance of a ‘shoulder’ on the faster-sedimenting side of especially the first peak in the polysome region, which likely reflects accumulation of ‘half-mers’, i.e. polysomes containing one (or more) complete ribosomes plus a 40S subunit, reflecting the shortage of 60S subunits. At later times, there was a further loss of polyribosomes and a striking further increase in ‘half-mers’ (Figure 3C and Supplementary Figure S3F).

The mRNAs for RPs contain 5′-terminal tracts of oligopyrimidines (5′-TOPs), which confer positive regulation by mTORC1 (37,38). We analysed the polysomal association of two 5′-TOP mRNAs for components of the translational machinery, eEF1A and RPL11 and the β-actin mRNA (a translationally efficient, non-5′-TOP mRNA) (Figure 3C). All three were associated with polysomes under control conditions (RPL11 with smaller ones than the other two, reflecting its short coding region) and remained associated with the polysomal region of the profile after induction of BOP1Δ, rather than appearing in the non-translated fraction at the top of the gradient, despite the overall decrease in polysome levels. This shows that the recruitment of ribosomes on to 5′-TOP mRNAs remains active under these conditions (Figure 3C). This is consistent with the enhanced activity of mTORC1 signalling observed in cells expressing BOP1Δ and the well-known role of mTORC1 signalling in driving 5′-TOP mRNA translation. In line with the idea that mTORC1 signalling maintains the recruitment of ribosomes on to these 5′-TOP mRNAs in cells expressing BOP1Δ, PP242 caused them to shift markedly out of polysomes (Figure 3D). The continued polysomal association of the 5′-TOP mRNAs in BOP1Δ-expressing cells contrasts with the behaviour of other mRNAs, such as those for the pro-survival proteins Mcl1 and Bcl2, which shifted towards smaller polysomes (or non-polysomal fractions) under this condition (Figure 3D). Thus, the continued association of 5′-TOP mRNAs with ribosomes is not a general feature of all mRNAs under this condition.

Even after long-term (72 and 96 h) BOP1Δ induction, the 5′-TOP mRNAs for eEF1A, RPS19 and RPL11 remained associated with multiple ribosomes to a greater extent than in control cells, whereas the non-TOP mRNAs for HnRNP A3 and β-actin tended to shift slightly away from this region of the profile (Supplementary Figure S4A and B). Serum starvation normally causes a shift of 5′-TOP mRNAs out of polysomes (39). In cells expressing BOP1Δ, in contrast, the 5′-TOP mRNA for eEF1A remained associated with polysomes (more than in control cells) after serum-starved cells (Supplementary Figures S4B and S5A shows that BOP1Δ also induces eEF2 dephosphorylation in serum-starved cells). The increased polysomal association of 5′-TOP mRNAs in cells expressing BOP1Δ likely reflects the activation of mTORC1 signalling in them. This effect may again represent a cellular attempt to compensate for impaired ribosome biogenesis, but one that is ultimately ineffective given the deficiency in mature rRNA molecules and therefore in active ribosomes.

Interestingly, analysis of the ribosomal pellet from the cytoplasm of cells expressing GST-BOP1 or myc-BOP1Δ indicated that BOP1/BOP1Δ interacts with ribosomes (Supplementary Figure S3D and E). Fractionation of lysates from cells expressing BOP1Δ on sucrose density gradients revealed that some BOP1Δ is associated with polysomal region (Supplementary Figure S3F).

### Addressing the mechanism by which mTORC1 is activated after disruption of rRNA production

It is well-established that mTORC1 signalling can be activated by PKB via phosphorylation of TSC1/2 (15). However, expression of BOP1Δ or knock-down of WDR12 did not affect the phosphorylation of PKB (Figures 1E and 2A and B). Expressing BOP1Δ did cause a modest increase in the phosphorylation of ERK (Figure 2E), signalling through which can activate mTORC1 [see, e.g. (40)]. Nonetheless, complete blockade of this pathway using AZD6244 did not prevent the BOP1Δ-induced phosphorylation of S6 (Figure 2E), although it did affect the phosphorylation of eEF2, indicating an input from this pathway, perhaps via p90^RSK^ (24). Under some conditions, mTORC1 can be regulated by the ‘stress-activated’ p38 MAP kinase pathway (41,42). Induction of BOP1Δ did not increase the phosphorylation of p38 MAPK (Supplementary Figure S3G), although it was enhanced by treating cells with sodium arsenite, used as a ‘positive control’ (data not shown).

**Figure 4.**
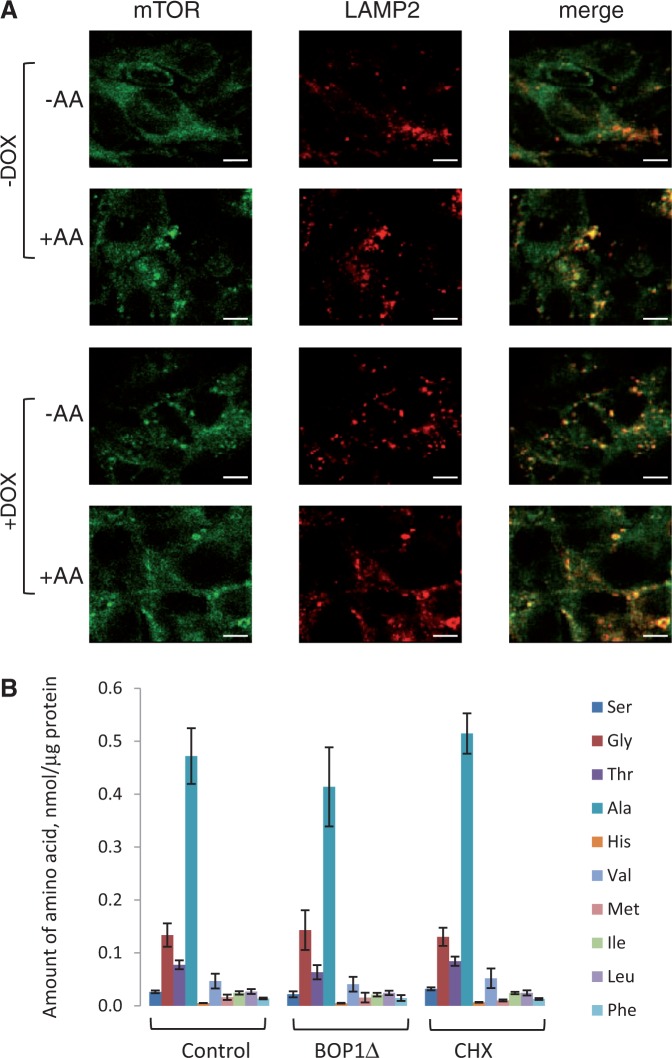
Expression of BOP1Δ affects the localization of mTOR but not intracellular amino acid levels. (**A**) Cells inducibly expressing myc-BOP1Δ were cultured in complete medium with/without doxycycline for 48 h, and then without serum in presence (+AA) or absence (−AA) of amino acids for 1 h. Analysis by immunofluorescence staining used mTOR (green) and LAMP2 (red) antibodies. Yellow dots represent co-localization. Scale bar, 10 μm. (**B**) Cells inducibly expressing myc-BOP1Δ were cultured in complete medium with/without doxycycline for 48 h. Cells were then lysed in ice-cold H_2_O, deproteinized and dried. Amino acid levels were determined by high performance liquid chromatography.

Activation of mTORC1 requires its amino acid-induced association with lysosomes (43). Importantly, induction of BOP1Δ increased the localization of mTOR to lysosomes, as assessed using the lysosomal marker LAMP2 (merged image, Figure 4A), especially in the absence of amino acids. Amino acid starvation caused a marked translocation of mTOR from lysosomes to the cytoplasm in control cells (Figure 4A), confirming these cells respond to amino acid starvation as expected. Our data suggest that BOP1Δ expression promotes mTORC1 signalling by inducing the localization of mTOR to a cellular locale where it is known to be activated. It has been shown previously that Rag proteins are critical for the lysosomal localization of mTORC1 and its activation (43,44). To study the role of the Rags in the control of mTORC1 signalling in response to expressing BOP1Δ, we overexpressed either constitutively active Rags (RagB[Q99L]/RagC[S75L] or inactive Rag mutants (RagB[T54L]/RagC[Q120L]) (43,44); their expression was verified by western blot (Supplementary Figure S6A). We then used immunofluorescence to study the localization of mTOR in the absence or presence of the expression of BOP1Δ and with/without amino acids. Co-localization was assessed using Costes’ method [see (45)]. This confirmed that BOP1Δ enhanced the association of mTOR with lysosomes (Supplementary Figure S6B). As expected, the expression of the active Rag mutants increased the lysosomal levels of mTOR, even in the absence of amino acids, and BOP1Δ caused a further increase (Supplementary Figure S6C). The inactive Rag mutants decreased lysosomal mTOR, even when amino acids were present (Supplementary Figure S6D), but did not prevent the increase caused by BOP1Δ, although they did appear to blunt this effect somewhat. This implies that the effect of BOP1Δ on the localization of mTOR is not dependent on active Rags, and suggests additional mechanisms are involved here.

In addition, expressing BOP1Δ did not significantly affect intracellular levels of any amino acids tested, including the branched-chain amino acids, which exert the greatest effects on mTORC1 (Figure 4B) (46), perhaps because the cells are in medium replete with amino acids, so that intracellular levels are already relatively high. Inhibiting protein synthesis with cycloheximide also did not further enhance amino acid levels, in contrast to the situation in amino acid-starved cells (47). Furthermore, because the activation of mTORC1 caused by expressing BOP1Δ is still stimulated by amino acids (Supplementary Figure S1B), localization to lysosomes is insufficient to activate mTORC1 in this setting and an additional input from amino acids appears to be needed.

### Expression of BOP1Δ promotes the inactivation of eEF2K

The decreased phosphorylation of eEF2 caused by interfering with rRNA processing likely reflects the fact that mTORC1 signalling is activated under these conditions and that this pathway negatively regulates eEF2K, e.g. via S6K1 (24,48) combined with the decrease in eEF2K protein (Figure 1H). To test whether eEF2K is an inherently unstable protein and/or whether its stability is decreased by expressing BOP1Δ, we examined endogenous eEF2K levels in control or BOP1Δ-expressing cells during treatment for various times with cycloheximide. At all times tested, eEF2K levels were lower in cells expressing BOP1Δ than controls, but did not decline over 120 min under either condition (Supplementary Figure S5B), indicating eEF2K is not intrinsically unstable or destabilized following BOP1Δ induction.

**Figure 5.**
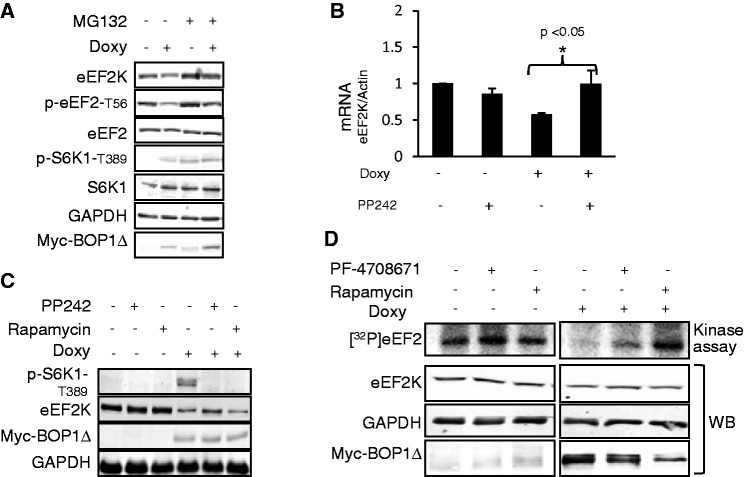
Regulation of eEF2K in cells expressing BOP1Δ. (**A**) T-REx cells were treated for 15 h with 1 µg/ml doxycycline and then for 1 h with 20 µg of MG132. Cell lysates were subjected to western blot. (**B**) T-REx cells were treated for 15 h with 1 µg/ml doxycycline and treated with PP242 (1 µM, 15 h). Total RNA was extracted and subjected to RT-qPCR analysis for eEF2K and β-actin mRNAs. The significance was determined by *t*-test. (**C**) T-REx cells expressing BOP1Δ were cultured in complete medium with/without doxycycline. After 33 h, in some cases, cells were treated with 1 µM PP242 or 100 nM rapamycin for 15 h. Cells were lysed and 20 µg of lysate proteins were used for western blot analysis. (**D**) T-REx cells inducibly expressing BOP1Δ were cultured in complete medium with/without doxycycline. After 36 h, in some cases, cells were treated overnight with 10 µM PF-4708671 or 100 nM rapamycin. Cells were lysed and 20 µg of protein were used for western blot analysis or to measure the activity of eEF2K using eEF2 and [γ-^32^P]ATP as substrates.

The proteasome inhibitor MG132 prevented the BOP1Δ-induced decrease in total eEF2K (Figure 5A), and also enhanced eEF2K levels in control cells, consistent with an earlier report that eEF2K levels can be regulated via the proteasome (49). Although MG132 did blunt the decrease in eEF2 phosphorylation caused by inducing BOP1Δ, it did not prevent it, confirming that additional regulatory events, which probably repress eEF2K activity, are also involved (Figure 5A). Analysis of the levels of the eEF2K mRNA revealed that BOP1Δ expression led to a fall in this mRNA, which was reversed by PP242 after overnight treatment (Figure 5B), indicating that the mTOR signalling can negatively control eEF2K mRNA expression, at least under these conditions. However, PP242 only slightly reversed the decrease in eEF2K protein levels at longer times (Figure 5C). This may indicate that additional mechanisms regulate the level of eEF2K itself or simply the ability of PP242 to inhibit protein synthesis (impairing the recovery in eEF2K protein levels).

We next used specific signalling inhibitors to further explore the mechanisms underlying the control of S6K1 and eEF2K in cells expressing the BOP1Δ truncation mutant. To test the role of S6K in the control of eEF2K/eEF2 in this setting, we used the S6K inhibitor PF-4708671 (50). This compound blocked the BOP1Δ-induced phosphorylation of RPS6 and also promoted the phosphorylation of eEF2 (Figure 2E), confirming that S6K1 provides a link between mTORC1 and eEF2K here. However, it did not prevent the partial loss of eEF2K protein (Figure 2E) and only partially reversed the decrease in eEF2K activity seen in cells expressing BOP1Δ (Figure 5D). [As reported earlier (50), this compound tends to enhance the phosphorylation of S6K1; Figure 2E]. To assess whether other effects downstream of mTOR might be involved in regulating eEF2K here, we tested the effects of rapamycin and the mTOR kinase inhibitor PP242 (11). At short treatment times (1 h), rapamycin reversed the BOP1Δ-induced decrease in p-eEF2, but did not affect the eEF2K protein level (Figure 2E). Even at longer times (16 h), only PP242, and not rapamycin, caused a small increase in the levels of eEF2K protein and a larger rise in *EEF2K* mRNA (Figure 5B and C). Given that BOP1Δ does not alter the phosphorylation of PKB at S473 (e.g. Figure 1E), this may involve rapamycin-insensitive functions of mTORC1 (12,13) rather than mTORC2.

Induction of BOP1Δ caused a profound inhibition of eEF2K activity (measured *in vitro*), which was much more marked than the decrease in eEF2K protein levels (Figure 5D). The S6K inhibitor PF-4708671 partially reversed this effect, whereas rapamycin overcame it completely (Figure 5D), likely reflecting additional links between mTORC1 and eEF2K activity (24,51).

## DISCUSSION

Ribosome biogenesis and protein synthesis are intimately linked, as the former provides the ribosomes required to catalyse the synthesis of new polypeptides and the latter provides the RPs that are essential for ribosome biogenesis. This study demonstrates that defects in the former process impact on signalling events that control mRNA translation. We show that impairing the transcription or processing of rRNA, by using low doses of actinomycin D or interfering with the PeBoW complex (by expressing BOP1Δ or knocking down its components), respectively, leads to the activation of S6K and the dephosphorylation of eEF2, a protein whose activity is indirectly regulated by S6K (22). Interference with rRNA production also led to decreased expression of eEF2K at both the mRNA and protein levels, providing an additional mechanism by which elongation can be stimulated. We also observe phosphorylation (i.e. inactivation) of the translational inhibitor protein, 4E-BP1, and concomitant disinhibition of eIF4E, a key component of the translation initiation machinery. These data show that human cells respond to these defects in rRNA production by activating a signalling pathway, mTORC1, which positively regulates both the initiation and elongation stages of mRNA translation. Because mTORC1 also positively regulates ribosome biogenesis at the levels of rRNA transcription and the translation of the 5′-TOP mRNAs that encode RPs, activation of mTORC1 may represent a concerted attempt by the cell to ‘rescue’ both mRNA translation and ribosome production in situations where the latter is defective. In both the settings we have studied, the attempt is actually fruitless because of the ongoing inhibition of rRNA synthesis (actinomycin D treatment) or impairment of the PeBoW complex, which causes a deficiency of 60S subunits. However, in physiological settings, this mechanism may well serve to help balance the production of rRNA and RPs, and to help cells compensate for deficits in ribosome biogenesis (see the scheme in Supplementary Figure S7).

Although previous work in yeast (52) has shown that deleting substrates for TORC1 that are involved in ribosome biogenesis (Sch9, a protein kinase and Sfp1) caused activation of TORC1 signalling, the underlying mechanism remained unclear. Our data provide the first evidence that impaired ribosome biogenesis elicits an analogous response in mammalian cells. It is important to emphasize that here we observe activation of mTORC1 signalling (i) on interfering specifically with rRNA production in distinct ways and (ii) without deleting substrates for mTORC1, which had already been shown to elicit increased phosphorylation of other mTORC1 substrates (e.g. S6K is hyperphosphorylated in 4E-BP1/2 knockout cells (53), perhaps due to relaxed competition between them for binding to mTORC1. Here, we observe the activation of mTORC1 signalling independently of deleting substrates of mTORC1, under differing conditions, which have the common consequence of disrupting rRNA synthesis. Defects in rRNA synthesis or processing cause translocation of mTOR to lysosomes, where this complex can be activated by Rheb, suggesting a possible mechanism by which mTORC1 is activated under these conditions; however, despite our attempts to investigate them, the mechanisms underlying this and the ensuing activation of mTORC1 remain to be fully elucidated.

A further, potentially important, finding from our study is that although BOP1Δ can promote the association of mTOR with lysosomes, as can amino acids (43,44), the activation of mTORC1 signalling by BOP1Δ still requires the addition of amino acids. This implies that, at least in this situation, localization of mTOR on lysosomes is insufficient to stimulate mTORC1 signalling. Other recent data (54) also suggest that the control of mTORC1 by amino acids is more complicated than previously thought.

Our findings also reveal that eEF2K is regulated at the level of its mRNA by signalling downstream of mTOR, in addition to the well-established control of its enzymatic activity by mTORC1 (23).

BOP1 associates with nuclear (pre-)ribosomes (27); our data show it is also associated with cytoplasmic ribosomes, in common with some other proteins involved in ribosome biogenesis [e.g. Rli1p/ABCE1 (55–57) and eIF6 (58)]. Interestingly, increased dosage of the *BOP1* gene is associated with colorectal cancer and enhanced levels of BOP1 mRNA (59).

Importantly, our data show that the effects of interfering with the production (transcription or processing) of rRNA contrast with those of decreasing the availability of RPs, by RNA interference or due to Diamond Blackfan Anaemia, an inherited disorder caused by mutations in RP genes (60). These conditions cause defective ribosome synthesis but lead to impairment, rather than activation, of mTORC1 signalling [(61,62) and our unpublished findings]. Consistent with this, treatment of Diamond Blackfan Anaemia patients with leucine, which promotes mTORC1 signalling (46), can ameliorate their symptoms [see also (63)].

Overall, the present data further extend the repertoire of regulatory loops that impinge on ribosome biogenesis to reveal a new one that regulates the translational machinery and helps cells maximize the productivity of the existing ribosomes by activating initiation and elongation under conditions of ‘ribosome stress’.

## SUPPLEMENTARY DATA

Supplementary Data are available at NAR Online.

## FUNDING

Project Grants (to C.G.P.) from the Ajinomoto Amino Acid Research Program; the British Heart Foundation [PG 11/18/28824]; and Cancer Research UK [C17397/A13576]. Funding for open access charge: British Heart Foundation, Cancer Research UK.

*Conflict of interest statement*. None declared.

## Supplementary Material

Supplementary Data

## References

[1] Drygin D, Rice WG, Grummt I (2010). The RNA polymerase I transcription machinery: an emerging target for the treatment of cancer. Annu. Rev. Pharmacol. Toxicol..

[2] Mayer C, Grummt I (2006). Ribosome biogenesis and cell growth: mTOR coordinates transcription by all three classes of nuclear RNA polymerases. Oncogene.

[3] Montanaro L, Trere D, Derenzini M (2008). Nucleolus, ribosomes, and cancer. Am. J. Pathol..

[4] Pianese G (1896). Beitraege zur Histologie und Aetiologie der Carconoms. Beitr. Pathol. Anat. Allgem. Pathol..

[5] Brandenburger Y, Arthur JF, Woodcock EA, Du XJ, Gao XM, Autelitano DJ, Rothblum LI, Hannan RD (2003). Cardiac hypertrophy *in vivo* is associated with increased expression of the ribosomal gene transcription factor UBF. FEBS Lett..

[6] Lempiainen H, Shore D (2009). Growth control and ribosome biogenesis. Curr. Opin. Cell Biol..

[7] Iadevaia V, Zhang Z, Jan E, Proud CG (2012). mTOR signaling regulates the processing of pre-rRNA in human cells. Nucleic Acids Res..

[8] Gingras A-C, Raught B, Sonenberg N (2001). Regulation of translation initiation by FRAP/mTOR. Genes Dev..

[9] Jastrzebski K, Hannan KM, Tchoubrieva EB, Hannan RD, Pearson RB (2007). Coordinate regulation of ribosome biogenesis and function by the ribosomal protein S6 kinase, a key mediator of mTOR function. Growth Factors.

[10] Xiao L, Grove A (2009). Coordination of ribosomal protein and ribosomal RNA gene expression in response to TOR signaling. Curr. Genomics.

[11] Feldman ME, Apsel B, Uotila A, Loewith R, Knight ZA, Ruggero D, Shokat KM (2009). Active-site inhibitors of mTOR target rapamycin-resistant outputs of mTORC1 and mTORC2. PLoS Biol..

[12] Thoreen CC, Kang SA, Chang JW, Liu Q, Zhang J, Gao Y, Reichling LJ, Sim T, Sabatini DM, Gray NS (2009). An ATP-competitive mTOR inhibitor reveals rapamycin-insensitive functions of mTORC1. J. Biol. Chem..

[13] Wang X, Beugnet A, Murakami M, Yamanaka S, Proud CG (2005). Distinct signaling events downstream of mTOR cooperate to mediate the effects of amino acids and insulin on initiation factor 4E-binding proteins. Mol. Cell. Biol..

[14] Sarbassov DD, Guertin DA, Ali SM, Sabatini DM (2005). Phosphorylation and regulation of Akt/PKB by the rictor-mTOR complex. Science.

[15] Huang J, Manning BD (2008). The TSC1-TSC2 complex: a molecular switchboard controlling cell growth. Biochem. J..

[16] Mayer C, Zhao J, Yuan X, Grummt I (2004). mTOR-dependent activation of the transcription factor TIF-IA links rRNA synthesis to nutrient availability. Genes Dev..

[17] Hannan KM, Brandenburger Y, Jenkins A, Sharkey K, Cavanaugh A, Rothblum L, Moss T, Poortinga G, McArthur GA, Pearson RB (2003). mTOR-dependent regulation of ribosomal gene transcription requires S6K1 and is mediated by phosphorylation of the carboxy-terminal activation domain of the nucleolar transcription factor UBF. Mol. Cell. Biol..

[18] Grimm T, Holzel M, Rohrmoser M, Harasim T, Malamoussi A, Gruber-Eber A, Kremmer E, Eick D (2006). Dominant-negative Pes1 mutants inhibit ribosomal RNA processing and cell proliferation via incorporation into the PeBoW-complex. Nucleic Acids Res..

[19] Strezoska Z, Pestov DG, Lau LF (2000). Bop1 is a mouse WD40 repeat nucleolar protein involved in 28S and 5. 8S RRNA processing and 60S ribosome biogenesis. Mol. Cell. Biol..

[20] Chresta CM, Davies BR, Hickson I, Harding T, Cosulich S, Critchlow SE, Vincent JP, Ellston R, Jones D, Sini P (2010). AZD8055 is a potent, selective, and orally bioavailable ATP-competitive mammalian target of rapamycin kinase inhibitor with *in vitro* and *in vivo* antitumor activity. Cancer Res..

[21] Iadevaia V, Caldarola S, Tino E, Amaldi F, Loreni F (2008). All translation elongation factors and the e, f, and h subunits of translation initiation factor 3 are encoded by 5′-terminal oligopyrimidine (TOP) mRNAs. RNA.

[22] Wang X, Li W, Williams M, Terada N, Alessi DR, Proud CG (2001). Regulation of elongation factor 2 kinase by p90RSK1 and p70 S6 kinase. EMBO J..

[23] Proud CG (2007). Signalling to translation: how signal transduction pathways control the protein synthetic machinery. Biochem. J..

[24] Smith EM, Proud CG (2008). cdc2-cyclin B regulates eEF2 kinase activity in a cell cycle- and amino acid-dependent manner. EMBO J..

[25] Sanchez I, Hughes RT, Mayer BJ, Yee K, Woodgett JR, Avruch J, Kyriakis JM, Zon LI (1994). Role of SAPK/ERK kinase-1 in the stress-regulated pathway regulating transcription factor c-Jun. Nature.

[26] Fonseca BD, Smith EM, Lee VH, MacKintosh C, Proud CG (2007). PRAS40 is a target for mammalian target of rapamycin complex 1 and is required for signaling downstream of this complex. J. Biol. Chem..

[27] Rohrmoser M, Holzel M, Grimm T, Malamoussi A, Harasim T, Orban M, Pfisterer I, Gruber-Eber A, Kremmer E, Eick D (2007). Interdependence of Pes1, Bop1, and WDR12 controls nucleolar localization and assembly of the PeBoW complex required for maturation of the 60S ribosomal subunit. Mol. Cell. Biol..

[28] Dunlop EA, Dodd KM, Seymour LA, Tee AR (2009). Mammalian target of rapamycin complex 1-mediated phosphorylation of eukaryotic initiation factor 4E-binding protein 1 requires multiple protein-protein interactions for substrate recognition. Cell Signal..

[29] Baird FE, Beattie KJ, Hyde AR, Ganapathy V, Rennie MJ, Taylor PM (2004). Bidirectional substrate fluxes through the system N (SNAT5) glutamine transporter may determine net glutamine flux in rat liver. J. Physiol..

[30] Strezoska Z, Pestov DG, Lau LF (2002). Functional inactivation of the mouse nucleolar protein Bop1 inhibits multiple steps in pre-rRNA processing and blocks cell cycle progression. J. Biol. Chem..

[31] Pearson RB, Dennis PB, Han JW, Williamson NA, Kozma SC, Wettenhall REH, Thomas G (1995). The principal target of rapamycin-induced p70S6k inactivation is novel phosphorylation site within a conserved hydrophobic domain. EMBO J..

[32] Hara K, Yonezawa K, Weng Q-P, Kozlowski MT, Belham C, Avruch J (1998). Amino acid sufficiency and mTOR regulate p70 S6 kinase and eIF4E BP1 through a common effector mechanism. J. Biol. Chem..

[33] Horn HF, Vousden KH (2008). Cooperation between the ribosomal proteins L5 and L11 in the p53 pathway. Oncogene.

[34] Holzel M, Rohrmoser M, Schlee M, Grimm T, Harasim T, Malamoussi A, Gruber-Eber A, Kremmer E, Hiddemann W, Bornkamm GW (2005). Mammalian WDR12 is a novel member of the Pes1-Bop1 complex and is required for ribosome biogenesis and cell proliferation. J. Cell Biol..

[35] Loreni F, Thomas G, Amaldi F (2000). Transcription inhibitors stimulate translation of 5′ TOP mRNAs through activation of S6 kinase and the mTOR/FRAP signalling pathway. Eur. J. Biochem..

[36] Lam YW, Lamond AI, Mann M, Andersen JS (2007). Analysis of nucleolar protein dynamics reveals the nuclear degradation of ribosomal proteins. Curr. Biol..

[37] Meyuhas O, Dreazen A (2009). Chapter 3 Ribosomal Protein S6 Kinase From TOP mRNAs to Cell Size. Prog. Mol. Biol. Transl. Sci..

[38] Patursky-Polischuk I, Stolovich-Rain M, Hausner-Hanochi M, Kasir J, Cybulski N, Avruch J, Ruegg MA, Hall MN, Meyuhas O (2009). The TSC-mTOR pathway mediates translational activation of TOP mRNAs by insulin largely in a raptor- or rictor-independent manner. Mol. Cell. Biol..

[39] Jefferies HBJ, Reinhard G, Kozma SC, Thomas G (1994). Rapamycin selectively represses translation of the ‘polypyrimidine tract' mRNA family. Proc. Natl Acad. Sci. USA.

[40] Fonseca BD, Alain T, Finestone LK, Huang BP, Rolfe M, Jiang T, Yao Z, Hernandez G, Bennett CF, Proud CG (2011). Pharmacological and genetic evaluation of proposed roles of mitogen-activated protein kinase/extracellular signal-regulated kinase kinase (MEK), extracellular signal-regulated kinase (ERK), and p90RSK in the control of mTORC1 protein signaling by phorbol esters. J. Biol. Chem..

[41] Brenneisen P, Wenk J, Wlaschek M, Krieg T, Scharffetter-Kochanek K (2000). Activation of p70 ribosomal protein S6 kinase is an essential step in the DNA damage-dependent signaling pathway responsible for the ultraviolet B-mediated increase in interstitial collagenase (MMP-1) and stromelysin-1 (MMP-3) protein levels in human dermal fibroblasts. J. Biol. Chem..

[42] Popowski M, Ferguson HA, Sion AM, Koller E, Knudsen E, Van Den Berg CL (2008). Stress and IGF-I differentially control cell fate through mammalian target of rapamycin (mTOR) and retinoblastoma protein (pRB). J. Biol. Chem..

[43] Sancak Y, Bar-Peled L, Zoncu R, Markhard AL, Nada S, Sabatini DM (2010). Ragulator-Rag complex targets mTORC1 to the lysosomal surface and is necessary for its activation by amino acids. Cell.

[44] Sancak Y, Peterson TR, Shaul YD, Lindquist RA, Thoreen CC, Bar-Peled L, Sabatini DM (2008). The Rag GTPases bind raptor and mediate amino acid signaling to mTORC1. Science.

[45] Willett M, Brocard M, Davide A, Morley SJ (2011). Translation initiation factors and active sites of protein synthesis co-localize at the leading edge of migrating fibroblasts. Biochem. J..

[46] Kimball SR, Jefferson LS (2006). Signaling pathways and molecular mechanisms through which branched-chain amino acids mediate translational control of protein synthesis. J. Nutr..

[47] Beugnet A, Tee AR, Taylor PM, Proud CG (2003). Regulation of targets of mTOR (mammalian target of rapamycin) signalling by intracellular amino acid availability. Biochem. J..

[48] Redpath NT, Foulstone EJ, Proud CG (1996). Regulation of translation elongation factor-2 by insulin via a rapamycin-sensitive signalling pathway. EMBO J..

[49] Arora S, Yang JM, Hait WN (2005). Identification of the ubiquitin-proteasome pathway in the regulation of the stability of eukaryotic elongation factor-2 kinase. Cancer Res..

[50] Pearce LR, Alton GR, Richter DT, Kath JC, Lingardo L, Chapman J, Hwang C, Alessi DR (2010). Characterization of PF-4708671, a novel and highly specific inhibitor of p70 ribosomal S6 kinase (S6K1). Biochem. J..

[51] Browne GJ, Proud CG (2004). A novel mTOR-regulated phosphorylation site in elongation factor 2 kinase modulates the activity of the kinase and its binding to calmodulin. Mol. Cell. Biol..

[52] Lempiainen H, Uotila A, Urban J, Dohnal I, Ammerer G, Loewith R, Shore D (2009). Sfp1 interaction with TORC1 and Mrs6 reveals feedback regulation on TOR signaling. Mol. Cell.

[53] Le-Bacquer O, Petroulakis E, Paglialunga S, Poulin F, Richard D, Cianflone K, Sonenberg N (2007). Elevated sensitivity to diet-induced obesity and insulin resistance in mice lacking 4E-BP1 and 4E-BP2. J. Clin. Invest.

[54] Oshiro N, Rapley J, Avruch J (2013). Amino acids activate mTOR complex1 without changing Rag GTPase guanyl nucleotide charging. J. Biol. Chem..

[55] Dong J, Lai R, Nielsen K, Fekete CA, Qiu H, Hinnebusch AG (2004). The essential ATP-binding cassette protein RLI1 functions in translation by promoting preinitiation complex assembly. J. Biol. Chem..

[56] Kispal G, Sipos K, Lange H, Fekete Z, Bedekovics T, Janaky T, Bassler J, guilar Netz DJ, Balk J, Rotte C (2005). Biogenesis of cytosolic ribosomes requires the essential iron-sulphur protein Rli1p and mitochondria. EMBO J..

[57] Rodnina MV (2010). Protein synthesis meets ABC ATPases: new roles for Rli1/ABCE1. EMBO Rep..

[58] Miluzio A, Beugnet A, Volta V, Biffo S (2009). Eukaryotic initiation factor 6 mediates a continuum between 60S ribosome biogenesis and translation. EMBO Rep..

[59] Killian A, Sarafan-Vasseur N, Sesboue R, Le PF, Blanchard F, Lamy A, Laurent M, Flaman JM, Frebourg T (2006). Contribution of the BOP1 gene, located on 8q24, to colorectal tumorigenesis. Genes Chromosomes Cancer.

[60] Horos R, Von LM (2012). Molecular mechanisms of pathology and treatment in Diamond Blackfan Anaemia. Br. J. Haematol..

[61] Yip BH, Vuppusetty C, Attwood M, Giagounidis A, Germing U, Lamikanra AA, Roberts DJ, Maciejewski JP, Vandenberghe P, Mecucci C (2013). Activation of the mTOR signaling pathway by L-leucine in 5q- syndrome and other RPS14-deficient erythroblasts. Leukemia.

[62] Fumagalli S, Di CA, Neb-Gulati A, Natt F, Schwemberger S, Hall J, Babcock GF, Bernardi R, Pandolfi PP, Thomas G (2009). Absence of nucleolar disruption after impairment of 40S ribosome biogenesis reveals an rpL11-translation-dependent mechanism of p53 induction. Nat. Cell Biol..

[63] Boultwood J, Yip BH, Vuppusetty C, Pellagatti A, Wainscoat JS (2013). Activation of the mTOR pathway by the amino acid (L)-leucine in the 5q- syndrome and other ribosomopathies. Adv. Biol. Regul..

