# Supplementary material for: Impairing the production of ribosomal RNA activates mammalian target of rapamycin complex 1 signalling and downstream translation factors
Source: Nucleic Acids Res. 2014 Feb 13;42(8):5083–96. doi: 10.1093/nar/gku130 (PMC4005692; doi:10.1093/nar/gku130)
Supplement: Supplementary Data [file supp_42_8_5083__index.html]

Impairing the production of ribosomal RNA activates mammalian target of rapamycin complex 1 signalling and downstream translation factors — Impairing the production of ribosomal RNA activates mammalian target of rapamycin complex 1 signalling and downstream translation factors — Supplementary Data 

# Impairing the production of ribosomal RNA activates mammalian target of rapamycin complex 1 signalling and downstream translation factors

## Supplementary Data

files

**Files in this Data Supplement:**

- Supplementary Data - pdf file
