# Supplementary material for: Impairing the production of ribosomal RNA activates mammalian target of rapamycin complex 1 signalling and downstream translation factors
Source: Nucleic Acids Res. 2014 Feb 13;42(8):5083–96. doi: 10.1093/nar/gku130 (PMC4005692; doi:10.1093/nar/gku130)
Supplement: Supplementary Data [file supp_gku130_nar-03233-v-2013-File007.pdf]

SUPPLEMENTARY FIGURE LEGENDS

Figure S1. Effect of BOP1Δ on pre-rRNA processing and mTORC1 signaling. (A) HEK293 cells were transfected with 0.5μg HA-S6K1 DNA and different amounts of DNA encoding GST, GST-BOP1 or GST-BOP1Δ, as indicated. 48 h later, cells were harvested, and 20μg of lysate was analysed by western blots using the indicated antisera. (B) HEK293 cells were transfected with 0.5 μg HA-S6K1 DNA and 1μg GST-BOP1, GST-BOP1Δ or GST vector as indicated; 24 h later, cells were starved of serum for a further 15 h. In some cases, cells were starved of amino acids (60 min) prior to insulin stimulation (100 nM, 30 min). Cells were harvested and 20 μg of lysate was analyzed by western blot.

Figure S2. Effects of BOP1Δ on rRNA synthesis, cell number and Pol I promoter occupancy. (A) Cytoplasmic (C) and nuclear (N) distribution of myc-BOP1Δ in T-REx cells. Expression of BOP1Δ was induced by treating T-REx cells for 48 h with 1 μg/ml doxycycline for 15 h. Cytoplasmic and nuclear fractions were then prepared and analyzed by western blot. GAPDH (cytoplasmic) and lamin B (nuclear) were used as fractionation markers. The percentages of BOP1Δ in the C and N fractions are reported above the relevant lanes. Numbers show the quantification for the relative percentage based in the signal intensities, corrected for the relative volumes. (B) HEK293 cells were transfected with 0.5μg HA-S6K1 DNA and two different amounts of myc-BOP1Δ DNA as indicated. 48 h later, cells were harvested, and 20μg of lysate was analyzed by western blots using the indicated antisera. (C) T-REx cells were treated for 48 h with 1μg/ml doxycycline to induce expression of myc-BOP1Δ. Total RNA was extracted and subject to RT-qPCR analysis for p21 mRNA normalized to the total level of 28 S rRNA. (D) To assess effects on cell proliferation, T-REx cells were treated for 3 or 6 days with 1μg/ml doxycycline and were then counted in triplicate. Cell numbers versus days in culture are reported as a line graph and the data indicate the mean ± SD of triplicate samples. (E) Expression of BOP1Δ was e induced by treating T-REx cells for 15 h with 1 μg/ml doxycycline. An MTT assay was then carried out following the manufacturer's instructions. (F) A chromatin cross-linking and immunoprecipitation assay (ChIP) was performed in T-REx cells induced for 15 h with 1 μg/ml doxycycline. An antibody specific for UBF was employed in immunoprecipitation and associated DNA was quantitated by real-time qPCR. (G) HEK293 cells were transfected with scrambled siRNA or siRNA against BOP1 (20 nM) as indicated or treated with 20ng/ml with actinomycin D. Analysis by immunofluorescence staining used B23 (red) antibody and 4',6-diamidino-2-phenylindole (DAPI). The significance was determined by Student's t-test.

Figure S3. Effects of knocking down components of the PeBow complex on effectors of mTORC1 and mTORC2 and on ribosomal protein levels. (A-C) HEK293 cells were transfected with a scrambled siRNA or siRNAs against BOP1 or WDR12 (20 nM) as indicated; after 48 h, cells were harvested. Samples of cell lysate were subjected to western blots as indicated. The two isoforms of endogenous S6K (p70, p85) are indicated. In (C), two different siRNAs directed against BOP1 were used. In all cases, the phosphorylation of endogenous S6K was studied. (D) The T-REx cells were treated for 24 h with 1 μg/ml doxycycline and lysed and separated by ultracentrifugation into pellet (P, ribosomes) and post-ribosomal supernatant (S), and subjected to western blot analysis using the indicated antibodies. The percentages of BOP1Δ in the P and S fractions are reported above the relevant lanes. Numbers show the quantification for the relative percentage based in the signal intensities, corrected for the relative volumes. (E) HEK293 cells were transfected with 1μg DNA encoding GST-BOP1. 48 h later, lysates were prepared and subjected to

ribosome fractionation as in panel D. (F) Expression of BOP1Δ was induced by treating T-REx cells for 48 h with 1 μg/ml doxycycline Cell lysates were fractionated on sucrose-density gradients [the positions of so-called 'halfmers' are indicated']. The proteins in each fraction were precipitated by TCA (trichloroacetic acid) and analyzed by Western Blot. The proportion of each fraction applied to the gel was: fraction 1, 7%; 2, 14%; 3, 33% and 4-9, 50%. (G) Cells expressing BOP1Δ were cultured in complete medium with/without doxycycline for indicating time. Cells were lysed and 20 μg of lysate proteins were used for western blot analysis.

Figure S4. Polysomal association of mRNAs of interest in cells expressing BOP1Δ. (A,B) Expression of BOP1Δ was induced by treating T-REx cells with 1 μg/ml doxycycline for 3 (A) or 4 (B) days with 1 μg/ml doxycycline and then starved of serum overnight. The lysates were resolved on sucrose-density gradients and 9 fractions were collected. The RNA was extracted from each fraction and subjected to northern blot, membranes being hybridized with probes specific for eEF1A, β-actin, RPS7, RPS19, RPL11, BOP1 and HnRNPA3 mRNAs.

Figure S5. Effects of expressing BOPΔ on eEF2K. (A) T Expression of BOP1Δ was induced by treating T-REx cells for 48 h with 1 μg/ml doxycycline for.r 4 days. Cells were then starved of serum overnight, where indicated. Cell lysates were analyzed by western blot using the indicated antibodies. (B) Expression of BOP1Δ was induced by treating T-REx cells for 15 h with 1 μg/ml doxycycline. Cells were then treated for the indicated times with cycloheximide (10 μg/ml). Cell lysates were analyzed by western blot using antibodies specific for eEF2K and GAPDH.

Figure S6. Effects of Rag mutants on the BOP1Δ induced localisation of mTOR to lysosomes. Analysis by western blot of cell lysates from T-REx cells in some cases expressing myc-BOP1Δ (+ doxy) and were also transiently transfected with vectors encoding the constitutively-active Rag mutants (Rag B[Q99L] and Rag C[S75L]) or inactive Rags (Rag B[T54L] and Rag C[Q120L]). (B) Analysis by immunofluorescence staining used mTOR (green) and LAMP2 (red) antibodies in T-REx cells expressing myc-BOP1Δ.The cells were cultured in complete medium with/without doxycycline for 48 h. Cells were starved 1 h of serum and subsequently starved of amino acids for 50 minutes. Some cells, where indicated, were then stimulated for 10 min with amino acids (+AA). Cells were then fixed with 4% (v/v) para-formaldehyde for 10 min and permeabilized with PBS 0.1% Triton X100 for 5 min. Cells were rinsed 3 times with PBS and then incubated with PBS containing 0.5% (w/v) BSA for 1 h at room temperature. Primary antibodies, diluted 1/50 in PBS, were incubated at 4°C for 2 h. Secondary antibodies, diluted 1/200 in PBS, were incubated at room temperature for 1 h in the dark. (C) T-REx cells expressing myc-BOP1Δ and transient transfected with the constitutive active Rag mutants (Rag B[Q99L] and Rag C[S75L]) were treated as in panel B). (D) T-REx cells expressing myc-BOP1Δ and transient transfected with the constitutive inactive Rag (Rag B[T54L] and Rag C[Q120L]) were treated as in panel B). The co-localization between mTOR and LAMP2 was assessed using Costes' method.

Figure S7. Model.

Please see the Discussion for further details

Fig. S1

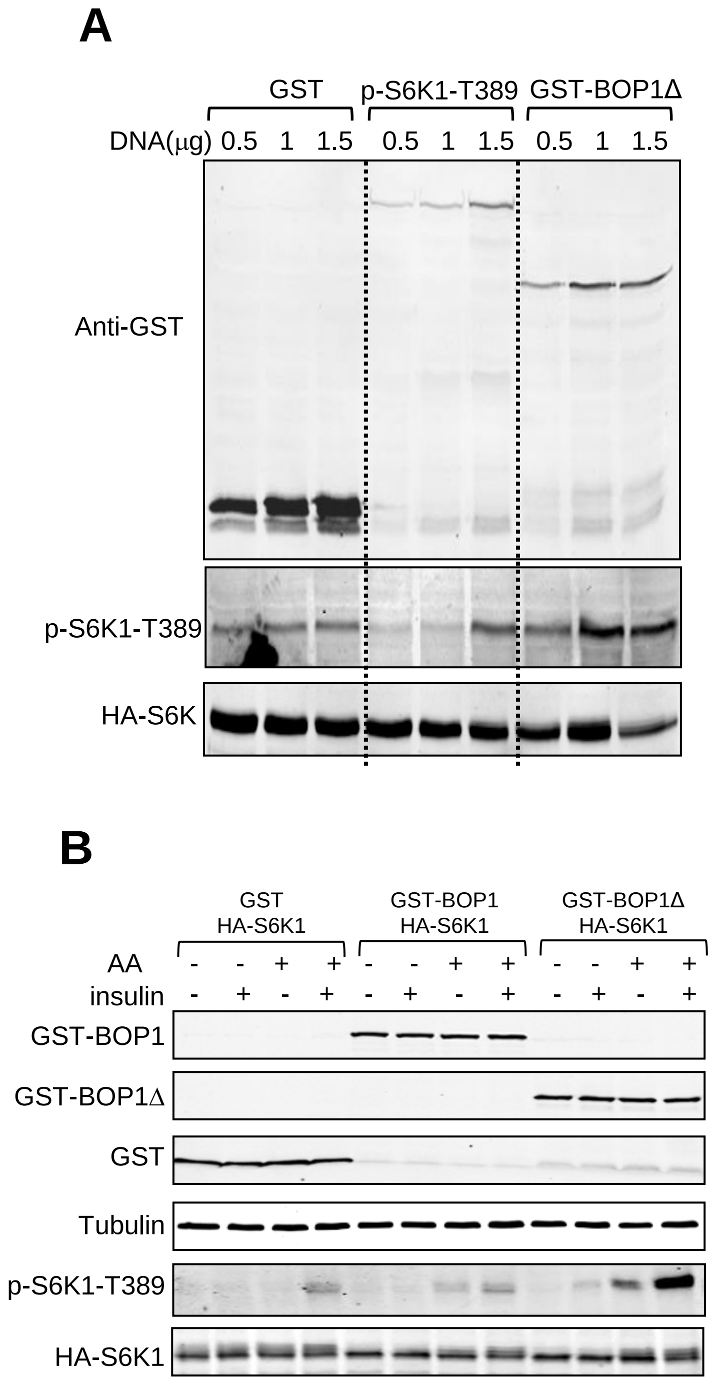
A
GST
p-S6K1-T389
GST-BOP1Δ
DNA(μg) 0.5 1 1.5 0.5 1 1.5 0.5 1 1.5
Anti-GST
p-S6K1-T389
HA-S6K
B
GST
HA-S6K1
GST-BOP1
HA-S6K1
GST-BOP1Δ
HA-S6K1
AA - - + + - - + + - - + +
insulin - + - + - + - + - + - +
GST-BOP1
GST-BOP1Δ
GST
Tubulin
p-S6K1-T389
HA-S6K1

Fig. S2

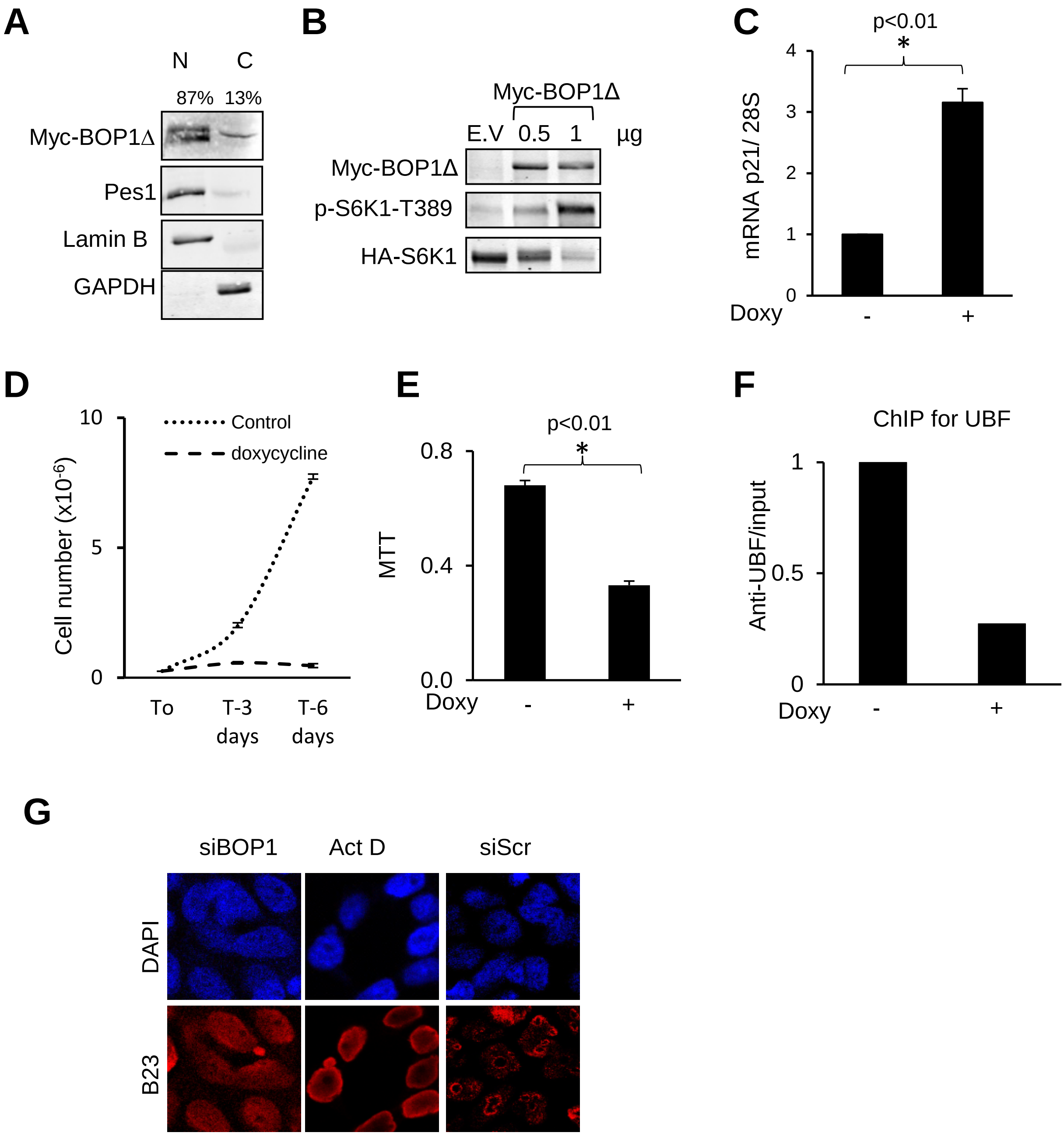
A
N
C
87%
13%
Myc-BOP1Δ
Pes1
Lamin B
GAPDH
B
Myc-BOP1Δ
E.V
0.5
1
µg
Myc-BOP1Δ
p-S6K1-T389
HA-S6K1
C
p<0.01
*
mRNA p21/ 28S
Doxy
-
+
D
Control
doxycycline
Cell number (x10-6)
To
T-3 days
T-6 days
E
p<0.01
*
MTT
Doxy
-
+
F
ChIP for UBF
Anti-UBF/input
Doxy
-
+
G
siBOP1
Act D
siScr
DAPI
B23

Fig. S3

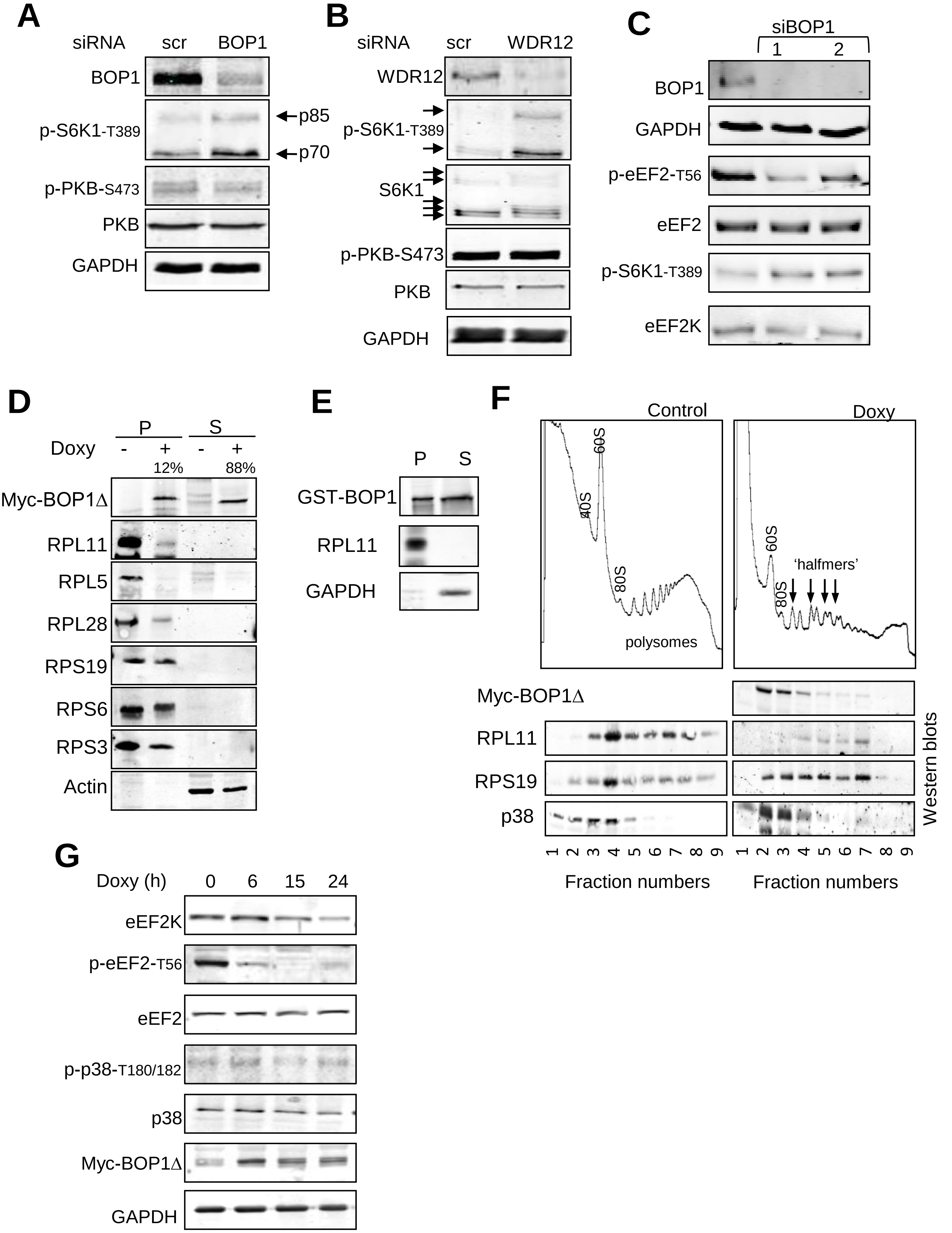

A
siRNA scr BOP1
BOP1
p-S6K1-T389
p85
p70
p-PKB-S473
PKB
GAPDH
B
siRNA scr WDR12
WDR12
p-S6K1-T389
S6K1
p-PKB-S473
PKB
GAPDH
C
siBOP1
1 2
BOP1
GAPDH
p-eEF2-T56
eEF2
p-S6K1-T389
eEF2K
D
P S
Doxy - + - +
12% 88%
Myc-BOP1Δ
RPL11
RPL5
RPL28
RPS19
RPS6
RPS3
Actin
E
P S
GST-BOP1
RPL11
GAPDH
F
Control
Doxy
60S
40S
80S
polysomes
'halfmers'
Myc-BOP1Δ
RPL11
RPS19
p38
Western blots
1 2 3 4 5 6 7 8 9
Fraction numbers
G
Doxy (h) 0 6 15 24
eEF2K
p-eEF2-T56
eEF2
p-p38-T180/182
p38
Myc-BOP1Δ
GAPDH

Fig. S4

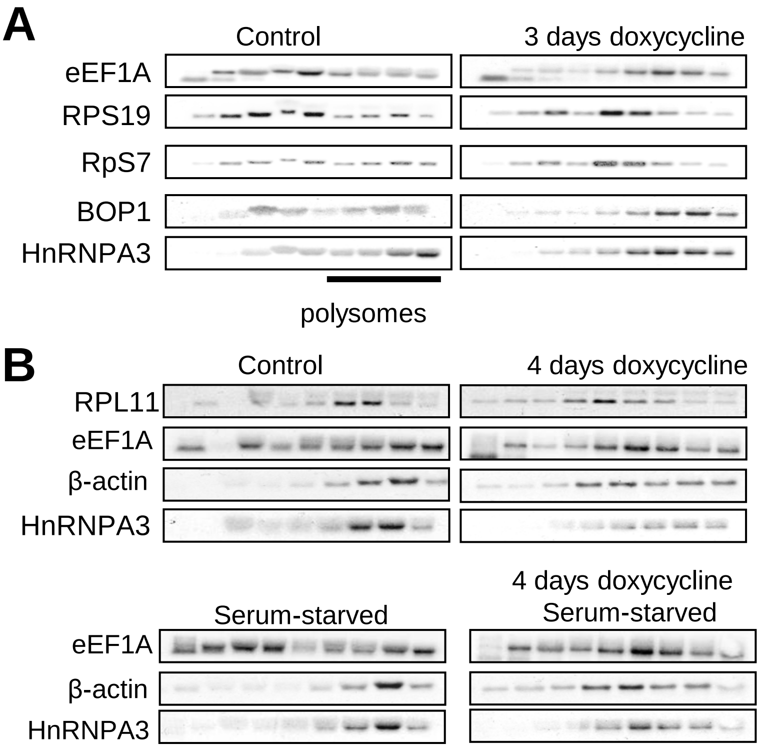
A
Control
3 days doxycycline
eEF1A
RPS19
RpS7
BOP1
HnRNPA3
polysomes
B
Control
4 days doxycycline
RPL11
eEF1A
β-actin
HnRNPA3
Serum-starved
4 days doxycycline
Serum-starved
eEF1A
β-actin
HnRNPA3

Fig. S5

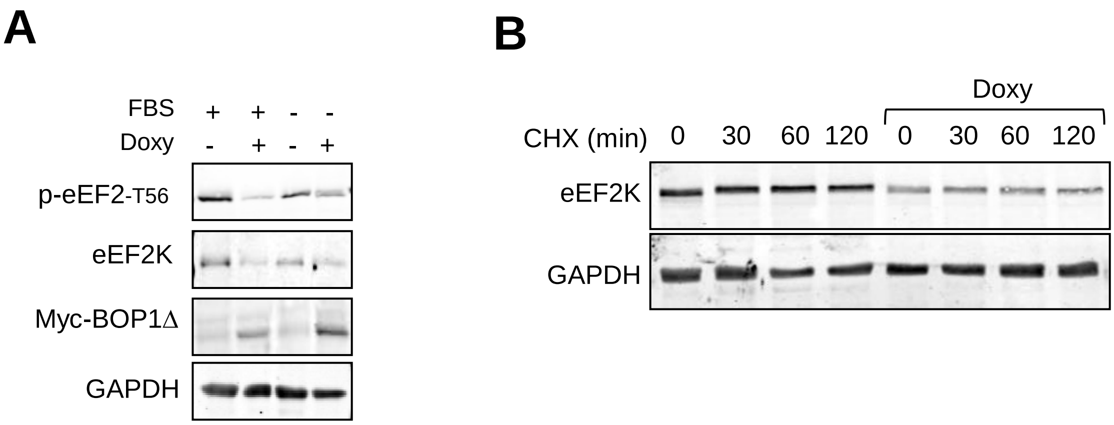
A
FBS + + - -
Doxy - + - +
p-eEF2-T56
eEF2K
Myc-BOP1Δ
GAPDH
B
Doxy
CHX (min) 0 30 60 120 0 30 60 120
eEF2K
GAPDH

Fig. S6

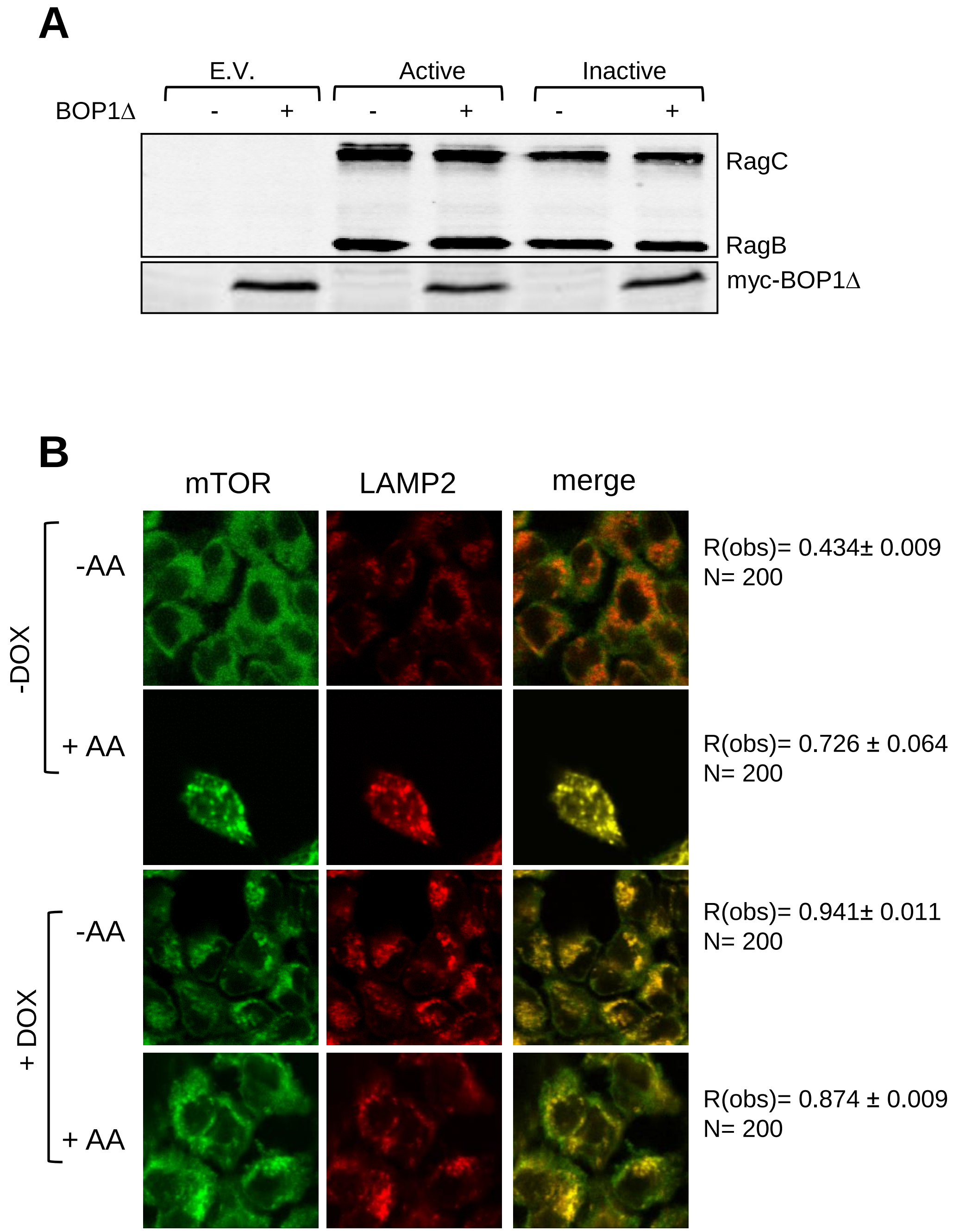
A
E.V.
Active
Inactive
BOP1Δ
-
+
-
+
-
+
RagC
RagB
myc-BOP1Δ
B
mTOR
LAMP2
merge
-DOX
-AA
+ AA
+ DOX
-AA
+ AA
R(obs)= 0.434± 0.009
N= 200
R(obs)= 0.726 ± 0.064
N= 200
R(obs)= 0.941± 0.011
N= 200
R(obs)= 0.874 ± 0.009
N= 200

Fig. S6

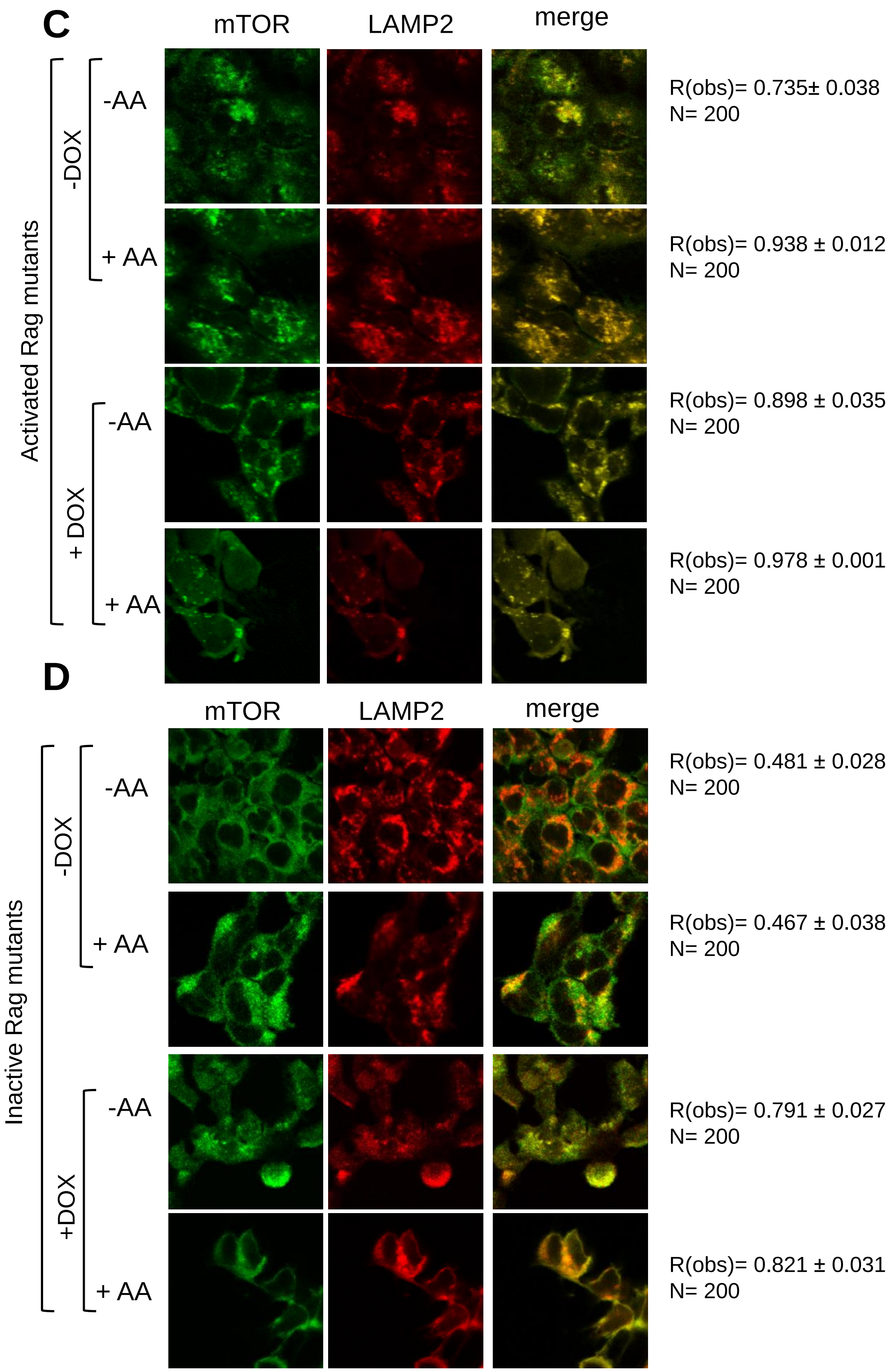
C
mTOR
LAMP2
merge
Activated Rag mutants
-DOX
+ DOX
-AA
+ AA
-AA
+ AA
R(obs)= 0.735± 0.038
N= 200
R(obs)= 0.938 ± 0.012
N= 200
R(obs)= 0.898 ± 0.035
N= 200
R(obs)= 0.978 ± 0.001
N= 200
D
mTOR
LAMP2
merge
Inactive Rag mutants
-DOX
+DOX
-AA
+ AA
-AA
+ AA
R(obs)= 0.481 ± 0.028
N= 200
R(obs)= 0.467 ± 0.038
N= 200
R(obs)= 0.791 ± 0.027
N= 200
R(obs)= 0.821 ± 0.031
N= 200

Fig. S7

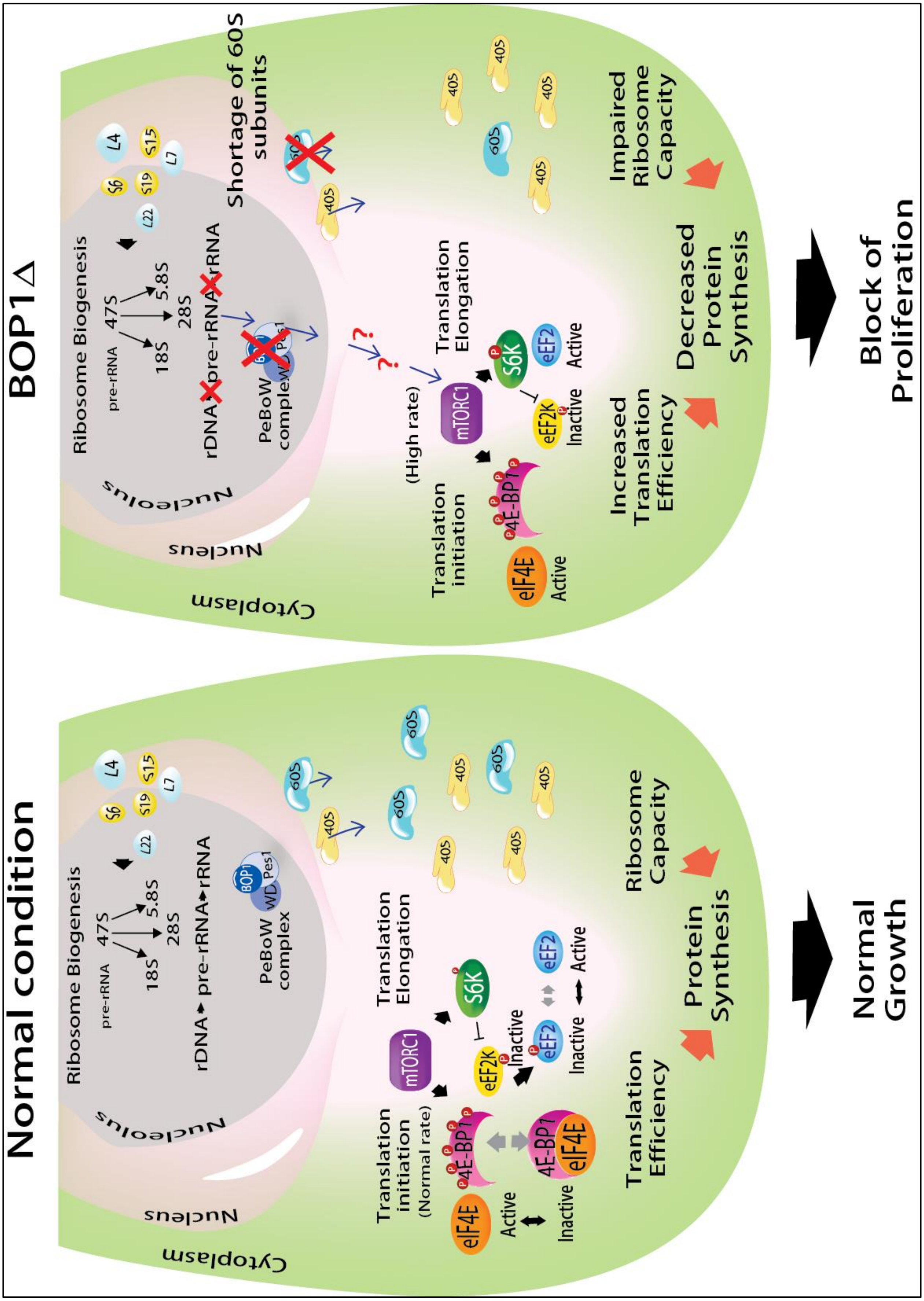

Normal condition
Ribosome Biogenesis
pre-rRNA
47S
18S
5.8S
28S
rDNA
pre-rRNA
rRNA
PeBoW complex
BOP1
WD
Pes1
Nucleolus
Nucleus
Cytoplasm
L4
S6
S19
S15
L7
L22
40S
60S
mTORC1
Translation initiation
(Normal rate)
Translation Elongation
4E-BP1
eIF4E
S6K
eEF2K
eEF2
Active
Inactive
Translation Efficiency
Protein Synthesis
Ribosome Capacity
Normal Growth
BOP1Δ
Shortage of 60S subunits
(High rate)
Increased Translation Efficiency
Decreased Protein Synthesis
Impaired Ribosome Capacity
Block of Proliferation
